# Extremely wet summer events enhance permafrost thaw for multiple years in Siberian tundra

**DOI:** 10.1038/s41467-022-29248-x

**Published:** 2022-03-23

**Authors:** Rúna Í. Magnússon, Alexandra Hamm, Sergey V. Karsanaev, Juul Limpens, David Kleijn, Andrew Frampton, Trofim C. Maximov, Monique M. P. D. Heijmans

**Affiliations:** 1 Plant Ecology and Nature Conservation Group, Wageningen University & Research, Wageningen, The Netherlands; 2 Department of Physical Geography, Stockholm University, Stockholm, Sweden; 3 Bolin Centre for Climate Research, Stockholm University, Stockholm, Sweden; 4 Institute for Biological Problems of the Cryolithozone, Siberian Branch of the Russian Academy of Sciences, Yakutsk, Russia

**Keywords:** Hydrology, Climate change, Cryospheric science

## Abstract

Permafrost thaw can accelerate climate warming by releasing carbon from previously frozen soil in the form of greenhouse gases. Rainfall extremes have been proposed to increase permafrost thaw, but the magnitude and duration of this effect are poorly understood. Here we present empirical evidence showing that one extremely wet summer (+100 mm; 120% increase relative to average June–August rainfall) enhanced thaw depth by up to 35% in a controlled irrigation experiment in an ice-rich Siberian tundra site. The effect persisted over two subsequent summers, demonstrating a carry-over effect of extremely wet summers. Using soil thermal hydrological modelling, we show that rainfall extremes delayed autumn freeze-up and rainfall-induced increases in thaw were most pronounced for warm summers with mid-summer precipitation rainfall extremes. Our results suggest that, with rainfall and temperature both increasing in the Arctic, permafrost will likely degrade and disappear faster than is currently anticipated based on rising air temperatures alone.

## Abstract (web summary)

Thawing permafrost releases carbon that serves as a positive feedback on climate warming. Here the authors experimentally demonstrate that rainfall extremes in the Siberian tundra increase permafrost thaw for multiple years, especially if rainfall coincides with warm periods.

## Introduction

Permafrost has been degrading rapidly and ubiquitously in response to Arctic warming^1–6^. Climate models suggest that 24% (RCP2.6) to 70% (RCP8.5) of near-surface permafrost may disappear by 2100. This could result in the release of tens to hundreds Gt carbon into the atmosphere, further enhancing climate warming^7^. Although highly responsive to air temperature^1,8,9^, permafrost degradation rates also depend on other climatic, soil physical, hydrological and vegetation related factors^10–14^. Rainfall is one such factor that has been associated with enhanced permafrost thaw in modelling and short-duration observational studies^15–18^. Arctic precipitation is anticipated to increase^19,20^ by up to 60% locally (RCP8.5) by 2100^20^ and to increasingly shift from snow to rain due to rising air temperatures^21^. Increased seasonal variability of precipitation, particularly in summer, implies increased occurrence of extreme rain events^20,22^. However, experimental data on how this affects permafrost are currently lacking. We set out to quantify the magnitude and duration of the effect of rainfall extremes on permafrost thaw in a field experiment to contribute to improved projections of future permafrost degradation.

Observational studies of the effects of rainfall extremes on permafrost soils have shown divergent effects of rain on soil thermal regimes^16–18,23–26^. On the one hand, relatively warm infiltrating rain can enhance thaw^16–18^ through the transport of heat in infiltrating rainwater into colder soils^15,18,24^ or by increasing soil thermal conductivity, enabling more heat to penetrate into the soil^24,27,28^. On the other hand, increased soil moisture resulting from rainfall can slow down the warming of cold permafrost soils^23,25,26^ as the energy required to warm the wetter soil (heat capacity) is increased and more energy (latent heat) is required for phase changes during freezing, thawing and evaporation^24,25,27,28^. The balance between these opposing warming and cooling effects may depend, among other factors, on air temperatures and seasonal timing^24^. Potential interactive effects between rising summer temperatures and changing precipitation patterns in the future Arctic are poorly quantified.

Lastly, it is conceivable that effects of extreme rainfall can persist over multiple years, for instance through increased soil ice contents in winters following extremely wet summers^17^ or structural alteration of the upper permafrost layer following enhanced seasonal thaw^29^. The magnitude and duration of potential carry-over effects of extreme rainfall are presently unknown.

We assessed the impact of one extremely wet summer on permafrost thaw over three summers in a controlled field irrigation experiment (10 irrigated, 10 control plots) in the north-eastern Siberian lowland tundra. This region is characterised by thick, ice-rich permafrost^10,30^ and a distinctly continental climate with warm summers^31^, with high potential for substantial permafrost degradation. The irrigation treatment (+100 mm) was set to mimic an extremely wet summer for this ecosystem (191 mm compared to 81 mm on average in June–August^32^). To explore the dependence of rainfall effects on air temperature and seasonal timing, we used a physically based numerical model accounting for necessary thermal and hydrological processes in permafrost regions (Advanced Terrestrial Simulator [ATS])^33^. We calibrated the model using field measurements and then conducted a model-based investigation of thaw depth under various rainfall and temperature scenarios.

## Results and discussion

### Field irrigation experiment

**Fig. 1:**
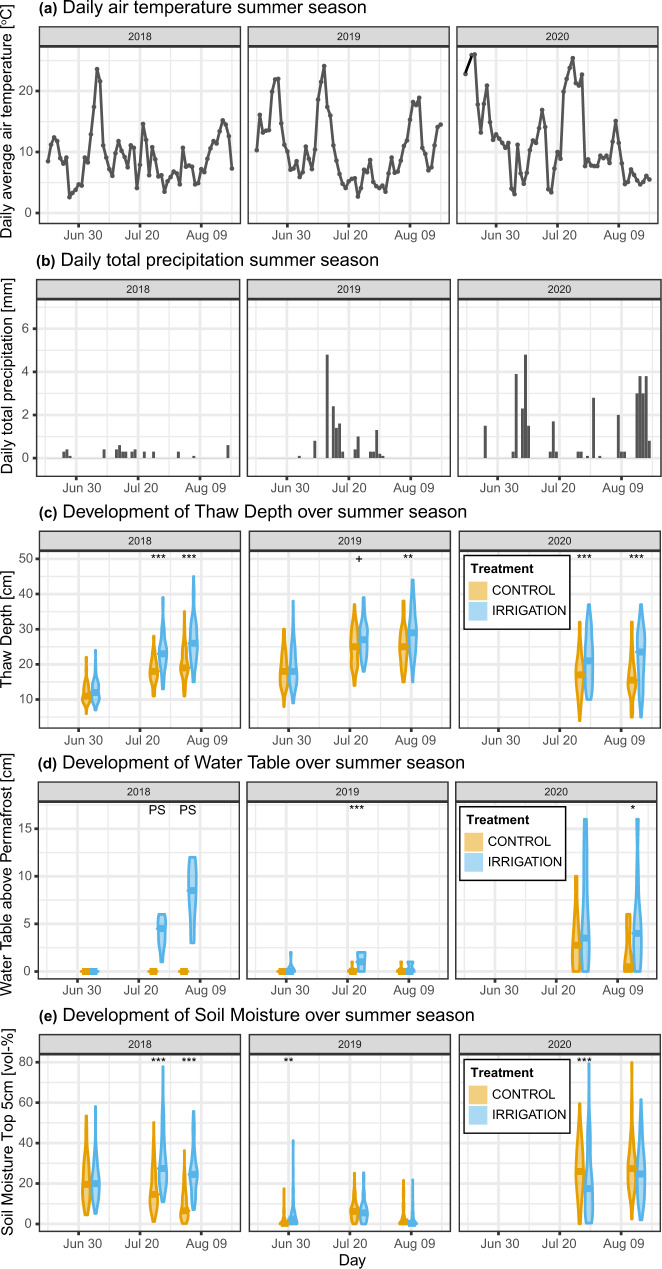
Results of the irrigation experiment. Abiotic conditions in control (orange) and irrigated (blue) plots during irrigation (2018) and subsequent summers without irrigation (2019 and 2020). The earliest 2018 measurements were taken before irrigation started. **a** Average daily air temperature and **b** total daily precipitation recorded in Chokurdakh (WMO station code 21649). **c** Thaw depth (*n* = 90 per violin). **d** Water table above permafrost in plot centres (*n* = 10 per violin). **e** Volumetric soil moisture content of the topsoil (5 cm depth) (*n* = 90 per violin). In **c**–**e**, violin length represents data range and violin width represents the probability density of the data distribution. Horizontal bars indicate group medians. Symbols above plots represent significant Tukey contrasts between irrigation and control per measurement date (+*p* < 0.1, *p < 0.05, **p < 0.01, ***p < 0.001). PS indicates perfect separation (see **d**), in which case no *p* values could be derived. Model specifications and estimated marginal means are in Supplementary Results I.

We found that extreme rainfall (+100 mm, +120%) increased permafrost thaw depth substantially over multiple years. During the summer of irrigation, thaw depths in irrigated plots gradually increased relative to control sites up to a 32% (+6.3 cm) difference in early August (Fig. 1c). The magnitude of this effect aligns with monitoring observations in Alaskan permafrost ecosystems, where a 10 mm increase in rainfall was estimated to result in a 0.7 cm increase in active layer thickness (ALT)^16^. In addition, extreme rainfall increased the volumetric moisture content of the topsoil relative to control plots following irrigation (Fig. 1e) and led to the formation of a water table above the permafrost (Fig. 1d). The following summers, thaw depths were still higher in irrigated plots than control plots in early August, with differences of 4.3 cm (+18%) in 2019 and 5.6 cm (+35%) in 2020. Warm temperatures during the years after irrigation (Fig. 1a) may have contributed to the sustained increase in thaw depth. Higher topsoil moisture in early summer 2019 and a continued increase in water tables on top of the permafrost (Fig. 1d, e) strongly suggest that added rainfall (partially) freezes up and is released in subsequent summers. Increased moisture content was observed in the topsoil as well as the subsoil (Supplementary Fig. 4) and is in line with earlier observational studies^17,34^. Apart from direct water input from irrigation, increased thaw depths in irrigated sites may cause lateral flow, reduced evaporation due to deeper infiltration or promoted the release of water from melting of excess ground ice^17^. The roles of evaporation and ground ice melt are unknown as they were not monitored during the experiment. No significant relations were found between microtopography, thaw depths and water tables, indicating that the roles of microtopography and lateral flow were likely limited compared to that of the irrigation treatment (Supplementary Fig. 19 and Supplementary Tables 12 and 13).

Substantial variation among observations, even within plots, suggests a high degree of spatial heterogeneity in thaw depths and soil moisture (Fig. 1c–e). Similarly, measurements varied among individual years. In the dry summer of 2018, no water tables were observed in control plots, whereas water tables in irrigated plots varied between 3 and 12 cm in early August. In 2019, soil moisture was very low in all plots and water tables were generally absent (Fig. 1e), likely caused by hot and dry meteorological conditions in early summer 2019 (Fig. 1a, b). In the wetter summer of 2020, higher water tables were observed, and topsoils in irrigated plots were drier than control plots, presumably related to the deeper thaw depth (Fig. 1c). Despite this spatio-temporal variability, irrigated plots still displayed more frequent and higher water tables and deeper thaw.

### Scenario analysis using numerical modelling

**Fig. 2:**
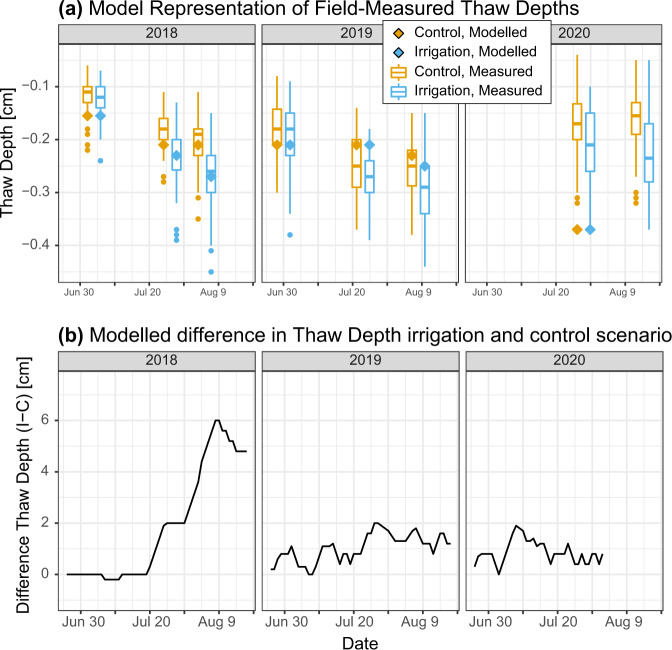
Model representation of the irrigation experiment. **a** Modelled and measured thaw depth for 2018–2020 with mean-field measurement values. Boxplots for measured thaw depths represent all individual measurements in irrigated (blue) and control (orange) plots (*n* = 90 per box). Centre lines represent the median, box limits represent upper and lower quartiles, whiskers represent 1.5 times the interquartile range and points represent outliers. **b** Modelled difference between irrigation and control scenario, smoothed with a 5-day moving average. Modelled data are only available up until the end of the meteorological record (31 July 2020).

A physically based numerical model (the Advanced Terrestrial Simulator, ATS v.088^33^) driven by site meteorological data was used to provide mechanistic insight in support of the field experiment. ATS was configured for local site conditions by using field measurements to set layer properties and boundary conditions. Parameters for which no field measurements were available (water retention evaluators and soil thermal parameters) were calibrated within a predefined range based on literature values for an accurate representation of thaw depth measurements from the irrigation experiment (Supplementary Methods III and IV). Modelled thaw depth closely followed field-measured thaw depths across both scenarios representing control and irrigated plots during the year of irrigation, except in the extremely warm summer of 2020 (Fig. 2). Representation of site-measured soil temperature and moisture content, which were not used for model calibration, were generally accurate (Supplementary Figs. 7 and 8). Comparison of modelled thaw depths with historical field measurements from the same site^32,35^ indicate reasonable correspondence (Nash-Sutcliffe Efficiency [NSE] = 0.44, RMSE = 5.75 cm, Supplementary Fig. 9) and provides an independent test of model representation of local thaw dynamics. Modelled effects of irrigation on thaw depth were smaller than those observed in the field, indicating that the model-based results are conservative estimates.

Apart from differences in instantaneous thaw depths, the model yielded a 5 cm difference in maximum end-of-season thaw depth (or ALT) in 2018. Such an effect roughly corresponds to that of a 1.7 °C increase in mean summer temperature (Supplementary Fig. 18)^36^. Modelled ALT (control: 37 cm, irrigation: 42 cm) closely resembles typical ALT for this region^36^. An 8-day delay in complete freeze-up was modelled under irrigation compared to the control scenario (Supplementary Fig. 6a). Model results are in line with the experiment, showing sustained small increases in thaw depth of up to 2 cm in the irrigated scenario in 2019 and 2020 and a 2-day delay in freeze-up in 2019. This suggests that the model generally reproduces site thaw dynamics satisfactorily but yields conservative estimates of the effect of extreme rainfall.

Model results suggest that increased soil moisture after irrigation remained in the soil during autumn freeze-up, resulting in increased ice content throughout the soil profile in winter in irrigated sites (Supplementary Fig. 12). Subsequent release of soil moisture in following summers (Supplementary Figs. 6b and 8b) likely mediated the observed carry-over effect. Irrigation increased subsoil temperatures (Supplementary Fig. 6a) and thaw depth both directly through the input of heat from rainwater with a higher temperature than the subsoil (Supplementary Fig. 11) and indirectly through increased thermal conductivity of the wetter soil (Supplementary Fig. 12). In contrast, colder topsoils were observed under extreme rainfall, both in model results and field measurements (Supplementary Figs. 6a and 8a), as a result of evaporative cooling of the surface (Supplementary Fig. 10)^26,37^.

Model sensitivity analysis (Supplementary Results V) indicates that modelled rainfall effects on permafrost thaw vary with soil hydrological and thermal parameters and layer definition (thickness of organic and mineral soil layers) (Supplementary Fig. 13). In order to explore potential implications for permafrost thaw across permafrost environments, we extended our analysis by modelling effects of rainfall across a range of soil textural classes, variable peat layer thickness and several temperature scenarios. Within this range of simulations, enhanced thaw following rainfall increases was modelled across all soil textural types. Larger rainfall effects were modelled for models with shallower peat layers, warmer summer temperatures and soil parameterisations representing coarser mineral soil texture (Supplementary Fig. 14). Although this suggests that fine-textured soils do not show above-average susceptibility to rainfall effects (Supplementary Fig. 14), we did find clear effects of rainfall in our experimental sites, characterised by fine-textured soils (Supplementary Table 11). Likely, high summer temperatures during the experiment and the summers after (Fig. 1a) contributed to the substantial field-measured effect of rainfall on thaw depth.

**Fig. 3:**
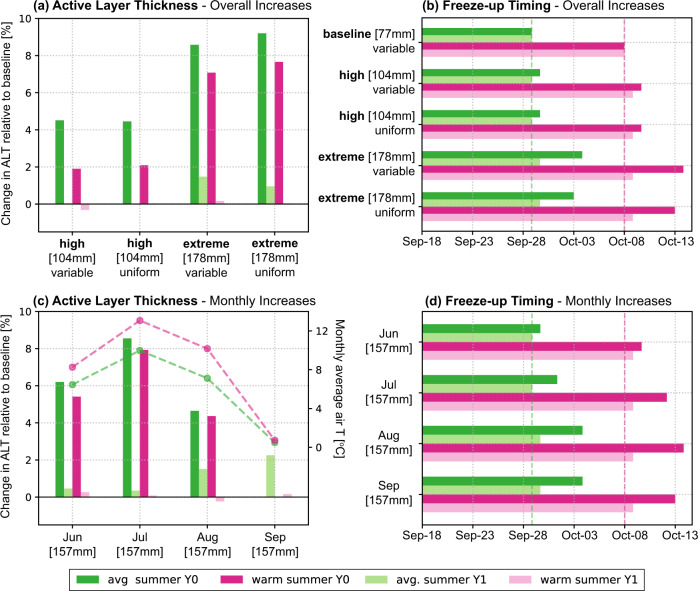
Effect of rainfall and temperature scenarios on modelled thaw dynamics. Effect of rainfall scenarios on active layer dynamics in average (green) and warm (pink) summers, for the year of altered rainfall (Y0, dark shades) and the year after (with baseline conditions) (Y1, light shades). **a** Modelled percentual difference in active layer thickness (ALT) under increased June–August (JJA) rainfall, with either uniform increase of JJA rainfall or variable increase (modelled to follow observed frequency-intensity distribution). **b** Modelled timing of complete freeze-up of the soil column under increased JJA rainfall. **c** Modelled percentual difference in ALT under increased rainfall in particular months. Dashed lines indicate mean temperature during the month of rainfall addition in average (green) and warm (pink) summers. **d** Modelled timing of complete freeze-up under increased rainfall in particular months. Changes (%) in ALT (**a**, **c**) are reported relative to baseline rainfall under the corresponding temperature scenario (average or warm summer). Positive changes percentages indicate increased ALT.

The calibrated model was used to analyse ALT and freeze-up timing under rainfall and temperature scenarios based on site meteorological data (Supplementary Fig. 3). The magnitude of ALT increase under increased rainfall depended on the timing of rainfall events relative to air temperature dynamics (Fig. 3c). Under average summer temperature (*T*_avg,JJA_ = 7.9 °C), baseline ALT was 27.2 cm, while extreme rainfall increased it to 29.6 cm. In a warm summer (*T*_avg,JJA_ = 10.6 °C), ALT was 46.6 cm under baseline conditions and 50.3 cm under extreme rainfall, indicating a larger net increase in warm summers. For high and extreme rainfall scenarios, no substantial differences were evident when additional rainfall was distributed uniformly over summer (JJA), compared to scenarios where additional rainfall was added following observed frequency-intensity distribution of daily total precipitation at the site (Supplementary Table 4). July rainfall extremes had the largest effect on ALT, whereas rainfall extremes in early and late summer had a smaller effect. Generally, the largest ALT increases were observed when high temperatures and high rainfall coincided, regardless of whether increases were uniform or variable (Supplementary Fig. 15). This suggests that the effect of rainfall strongly depends on its timing and is largest during warm conditions.

Rainfall extremes in September did not affect ALT in the same year because freeze-up had already started in September (Supplementary Figs. 16 and 17). However, August and September rainfall extremes have the strongest influence on freeze-up duration; the later rainfall is added to the system, the longer freeze-up is postponed. Delays of 6–7 days were found for the extreme rainfall scenario and August and September rainfall scenarios both in average and in warm summers (Fig. 3b, d). This is likely explained by higher heat capacity and increased release of latent heat in wetter soils during freezing, both of which delay autumn freeze-up^18,24^. These results indicate that rainfall extremes not only lead to a deeper thaw, but also extend the period over which soils remain (partly) unfrozen.

**Fig. 4:**
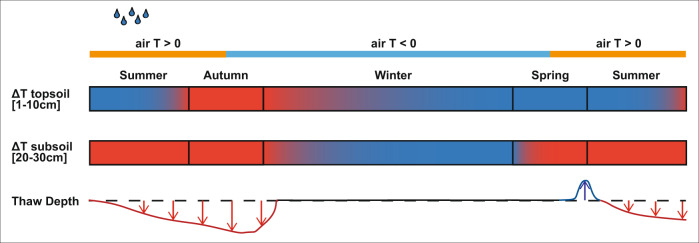
Conceptual diagram of the effect of extreme rainfall on the soil thermal regime. Schematic representation of the soil thermal regime under increased rainfall throughout the year and the following summer season, based on field observations and model results (Fig. 1 and Supplementary Figs. 8, 16 and 17). The top two bars represent temperature differences in the organic topsoil (0–10 cm depth) and mineral subsoil (20–30 cm depth) relative to a situation with average rainfall. Red colours indicate warmer temperatures under extreme rainfall and blue colours represent colder temperatures under extreme rainfall. The bottom line represents differences in thaw depth compared to a situation with average rainfall, with red downward arrows representing deeper thaw, blue upward arrows indicating shallower thaw and black colours representing no thaw depth (fully frozen soil). The effects of extreme rainfall vary by depth, with topsoils exhibiting evaporative cooling and persistent warming throughout autumn and early winter, whereas subsoils show warming in summer and cooling in winter.

Model results indicate that the effects of rainfall extremes vary among seasons and soil depths. Soil temperature changes were most evident in subsequent winters (Supplementary Figs. 16 and 17). Warming effects were visible in the topsoil in autumn and early winter due to delayed freeze-up, especially under late summer rainfall, which resulted in increases in topsoil temperature of up to 2 degrees during early winter. Modelled winter subsoil temperatures were lower than in the baseline scenario likely due to increased thermal conductivity under higher ice content (Supplementary Fig. 12). In spring, increased ice content impedes the warming of the topsoil through latent heat consumption (Supplementary Figs. 16 and 17). Similar changes in winter soil temperature were evident from field observations (Supplementary Fig. 8a). Soil temperatures in subsequent summers quickly returned to baseline conditions and only small differences in thaw depth persisted (Supplementary Figs. 16 and 17). Marginal delays in freeze-up persisted in subsequent summers, mostly under late summer and extreme rainfall and under warmer summer temperatures (Fig. 3b, d and Supplementary Figs. 16 and 17). The effects of extreme rainfall on the soil thermal regime in ice-rich Siberian lowland tundra are summarised in Fig. 4.

### Combined dynamics of summer rainfall and air temperature determine permafrost thaw dynamics

In recent years, enhanced permafrost thaw following extreme rainfall is being increasingly reported in observational and model-based studies^15–18^. Our study provides the first controlled, experimental estimates of the magnitude and duration of such effects. We found a substantial increase in thaw depth (up to 35%) under a 120% increase (+100 mm) in rainfall. Effects persisted for at least 2 years following the rainfall treatment. Soil thermal-hydrological modelling suggests that increased rainfall likely warmed the soil through direct input of warmer rainwater into colder soils (advective heat transfer) and increased heat conduction in summer. Rather than variability in rainfall per se, modelled effects of rainfall on ALT depended strongly on the timing of rainfall extremes relative to air temperature dynamics (Fig. 3 and Supplementary Fig. 15). Modelled increases in ALT were largest when rainfall extremes occurred during warmer mid-summer conditions (Fig. 3c and Supplementary Fig. 15). While the percentage increase in ALT was comparable in summers with average and high temperatures (Fig. 3a, up to 8.5%), net increases in ALT were larger under a combined increase in rainfall extremes and air temperature (up to 2.3 cm on average and 3.7 cm and warm summers) (Supplementary Fig. 15). These increases may be larger in reality, since the model yielded conservative estimates of the effects of irrigation on permafrost thaw (Fig. 2). Interactive effects with temperature may be attributed to increased heat transfer into the soil with infiltration of rainwater, since the temperature of rainwater tends to follow ambient air temperature^18^. Larger temperature gradients between soil and soil surface in warmer periods may also enhance conductive heat transport into the soil^24^. Late summer rainfall had little to no effect on ALT in the same season, but most pronouncedly delayed freeze-up and showed the highest potential for carry-over effects (Fig. 3c, d and Supplementary Figs. 16 and 17). As the frequency of extreme rainfall events in the Arctic is anticipated to increase^19^, it is important that rainfall effects on permafrost dynamics are accounted for in projections of future permafrost degradation. Our findings suggest that this requires high temporal resolution (ideally daily) climate data with an accurate representation of rainfall extremes and concurrent air temperatures, and a detailed representation of soil thermal hydrology and advective heat transfer from infiltrating rain in land surface models.

### Implications for thermal-hydrological modelling of permafrost soils

Using state-of-the-art numerical modelling of soil thermal hydrology, we were able to support field-observed effects with mechanistic insight into the effects of extreme rainfall on soil thermal dynamics. Our model parametrization represented field-measured permafrost thaw dynamics reasonably well (Fig. 2 and Supplementary Fig. 9). Still, modelled differences in thaw depth under increased rainfall and carry-over effects were conservative compared to those measured in the experimental treatments. This may be a result of overestimation of evaporative fluxes and resulting cooling (Supplementary Figs. 10 and 11). The model indicates substantial evaporative topsoil cooling (Supplementary Figs. 6a, 16 and 17), which was not as evident from field measurements (Supplementary Fig. 8a). Moreover, in its current configuration, ATS only accounts for surface evaporation using a vegetation-dependent roughness length^38^, and disregards potential further effects of vegetation (e.g., transpiration, retention of moisture in moss tissue and canopy shading)^12,35,39^. This may help explain the smaller effect of rainfall in model results compared to field results^15^. As future changes in Arctic vegetation are expected to alter the surface energy budget and thermal properties of permafrost soils^12^, expansion of soil thermal hydrology models to include canopy processes and transpiration is recommended.

Lastly, fine-scale landscape heterogeneity causes a wide range of soil hydrothermal properties, which can strongly control thaw dynamics and their response to climate change^4,12,13,36,40^. This was also evident from the fairly wide range of field-observed thaw depths, temperature and moisture conditions (Fig. 1c–e and Supplementary Figs. 4 and 5). Our one-dimensional numerical model only considers averaged site conditions and behaviour, leading to potential inaccuracies and a disregard for the lateral transport of moisture and heat and spatial heterogeneity of effects. Extension to 3D numerical models^40^ can be used to account for such nuances if sufficient spatially distributed field data are available.

### Implications for permafrost ecosystems

The identified impact of rainfall on permafrost thaw dynamics suggests increased ecosystem change and feedback to the climate in summers that are both warm and wet. With persistent Arctic warming and anticipated increases in rainfall extremes^20,22^, near-surface permafrost may degrade and disappear faster than is currently anticipated based on temperature changes alone (e.g., 40% areal loss under 2 °C of warming^8^). This may apply especially to ice-rich permafrost, where enhanced thaw following combined warming and rainfall extremes can result in soil subsidence due to the melting of abundant ground ice (thermokarst). Thermokarst triggers local feedbacks such as concentration of lateral flow, accumulation of water in the soil profile (Fig. 1d and Supplementary Fig. 6b) and accumulation of snow in depressions in winter, accelerating permafrost degradation over longer timescales^13,30,32,41^. Extreme summer drought, in contrast, may protect permafrost from high summer temperatures due to stronger thermal insulation of dry soil and reduced heat inputs from infiltration of rainwater.

Apart from the deeper thaw, model results indicate delayed autumn freeze-up following rainfall extremes. Methane emissions during freeze-up are considerably higher than those during winter and spring and may constitute as much as 20% of total annual methane emissions^42,43^. A delayed or prolonged freeze-up period in wetter soils thereby likely enhances methane emission^42^. This freeze-up delay was largest under late summer rainfall extremes (Fig. 3d). Due to temperature increases, an increasing proportion of autumn precipitation will fall as rain rather than snow in the future^20,21^, likely further contributing to autumn methane emissions. In addition, warmer and wetter conditions in subsoils (Supplementary Figs. 16 and 17) may promote methane production^18,44^, while methane oxidation may be reduced in wetter and colder (Supplementary Figs. 16 and 17) topsoils^37^. As a result, methane emissions may increase more than would be anticipated based on temperature increases alone. Conversely, cooling of the topsoil and soil wetting can reduce CO_2_ emissions through reduced ecosystem respiration^37^. However, rainfall extremes and associated cloudiness have also been observed to substantially reduce CO_2_ uptake in Arctic ecosystems^45^. Effects of cloudiness were not accounted for using our methodology as irrigation was performed regardless of cloud cover and rainfall scenarios were modified without adjusting incoming shortwave radiation.

Lastly, impacts of rainfall-induced active layer deepening may vary among and within ecosystems. Although active layer deepening following extreme rainfall was modelled across a range of soil parameterisations (Supplementary Fig. 14), the magnitude of rainfall effects likely additionally depends on vegetation characteristics, topography, permafrost properties, regional climate and interactions among these. On a regional scale, climatic variability can regulate the depth of seasonally unfrozen soil and the extent to which rainwater can infiltrate, which in turn affects soil moisture dynamics and the relative importance of physical properties of the topsoil and subsoil in soil thermal-hydrological processes (Supplementary Fig. 14). This may lead to non-linear responses of permafrost thaw depth to rainfall increases under variable soil stratigraphy and physical properties (Supplementary Figs. 13 and 14). Topographical variability governs lateral flow and accumulation of water and heat, leading to localised effects^18,46^. Lastly, vegetation can exert a strong control on the ground thermal regime^12,35^, while vegetation itself may be subject to change under increasing rainfall. For instance, soil wetting following thermokarst can promote shifts from shrub- to graminoid dominated systems^31,47^, changing the surface energy balance^12^. Moderate wetting however may promote shrub growth under previously water-limited conditions^48,49^. Persistent deepening of the active layer following increased rainfall can increase nutrient availability and rooting space, stimulating the growth of deeper-rooting graminoids^50^. However, it may also facilitate deeper infiltration and drainage of rainwater^4^, resulting in a drying effect on longer timescales (see for instance reduced topsoil moisture in 2020, Fig. 1e). Although evidence of adverse impacts of rainfall on permafrost thaw is emerging from across the Arctic, a complete perspective requires holistic monitoring of the effects of rainfall extremes across various Arctic ecosystems and climatic zones over longer time periods to better understand how environmental variability affects the sensitivity of permafrost to rainfall and temperature increases. Given the extensive need for input data and parameterisation in permafrost thermal-hydrological modelling, challenges remain in upscaling to a wider Arctic context and accounting for key sources of uncertainty. Our current model representation suggests that environments with shallow organic layers, coarser soil textures and warm summers may be especially prone to increased permafrost thaw following extreme rainfall. Further extension across gradients in climate, topography, hydrology and vegetation is necessary to properly assess spatial variability in the response of permafrost thaw to rainfall.

### Outlook

Using a controlled field experiment, we showed that extreme rainfall can enhance permafrost thaw for multiple years. Model analysis indicated that the magnitude of this effect depends on concurrent summer temperatures, with larger effects when rain falls during warm periods. Extension of our model representation to various soil textural and stratigraphical classes suggests that larger effects of rainfall on permafrost thaw may be observed in regions with shallow organic soil layers and coarse mineral soil texture. As the response of permafrost to increased rainfall likely varies across a wider range of environmental conditions with a high degree of local interaction, spatially distributed evidence is necessary to assess the sensitivity of permafrost to future rainfall increases on a panarctic scale. Our combination of field irrigation experiments, monitoring and permafrost modelling proved to be a valuable approach to do so.

## Methods

### Study site

**Fig. 5:**
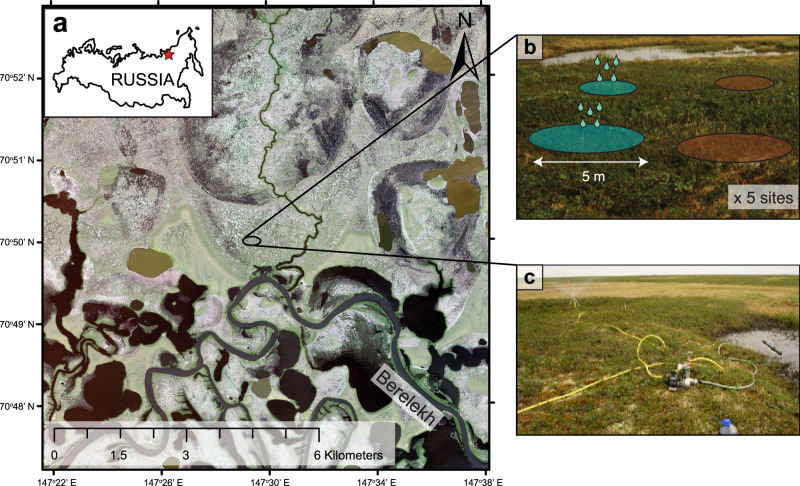
Study area and experimental setup. **a** Our study area is situated in the Indigirka lowlands at the Chokurdakh Scientific Tundra Station, in a drained thaw lake basin (alas) adjoining the floodplains of the river Berelekh. Image: WorldView-2 © 2019 Maxar Technologies. **b** In this alas, five sites were selected in shrub patches within 5–10 m distance from a thaw pond. In each site, four circular plots were set out: two irrigated plots (blue) and two control plots (brown) each with 5 m diameter. Plots were at least 5 m apart. **c** The irrigation system consisted of a filter tube and motor pump which simultaneously irrigated the two irrigated plots per site via hoses with a length of 10 m and sprinklers.

We studied the effects of increased rainfall on permafrost thaw dynamics in a drained thaw lake basin (or “alas”) in the “Kytalyk” Nature Reserve in the Indigirka Lowlands in north-eastern Siberia (70°49′N, 147°29′E) near the town of Chokurdakh (Fig. 5a). Such alases are representative of a major part of coastal north-eastern Siberia^51^. The area is characterised by a shallow active layer overlying ice-rich, continuous permafrost^52^. The mean annual temperature is −13.5 °C, with an average July temperature of 10.0 °C (1945–2019). The mean annual precipitation is 202 mm, of which 81 mm falls in summer (June, July and August) (1945–2019)^53,54^. Within the study area, elevated sites such as “Yedoma” ridges and pingos are characterised by tussock-sedge (*Eriophorum vaginatum*) and dwarf shrub vegetation. Lower elevation areas such as alases are characterised by slightly elevated shrub patches dominated by *Betula nana*, lichens and mosses, interspersed with waterlogged depressions characterised by aquatic species such as *Eriophorum angustifolium*, *Carex* spp. and *Sphagnum* spp^47,55^.

### Experimental design

We set out 20 circular plots of 5 m diameter in five clusters of four (two irrigation, two control), located in five dwarf shrub-dominated tundra patches in early summer 2018. Clusters were situated next to ponds that provided water for irrigation (Fig. 5b). Clusters were around 50–100 m apart and plots within clusters were at least 5 m apart. We installed a PVC well in the centre of each plot to monitor the water table. Prior to irrigation, we measured thaw depth and topsoil volumetric moisture content in nine locations per plot: eight points along the perimeter of the plot at 1 m distance from the plot edge, and one in the centre. We visually assessed cover of the main plant species and variation in microtopography and assigned plots to pairs within clusters based on similarity in thaw depth, water table, soil moisture, vegetation composition and microtopography. Plots from pairs were randomly assigned to irrigation and control, although in a few cases the length of the hoses of the irrigation system dictated the subdivision. No significant differences were evident in thaw depth, water table or soil moisture prior to irrigation (Supplementary Tables 5–10). Over the period of 6 July to 2 August 2018, we supplied 100 mm of irrigation to all irrigation plots and no irrigation to control plots using a motor pump (Fig. 5c). We set the amount of irrigation to mimic the extremely wet summer of 2011 (191 vs. 81 mm average in June–August^32^). The irrigation water supplied from ponds had a chemical composition similar to that of rainwater, and the temperature of irrigation water did not exceed that of the ambient air (Supplementary Methods I). We irrigated plots on an approximately biweekly basis in amounts of 10 or 15 mm with an application rate of 25 mm per hour. During the summers of 2018, 2019 and 2020, we repeated measurements of thaw depth, water table and topsoil volumetric moisture content at regular intervals. In eight plots (four irrigation, four control), we installed temperature and moisture loggers at 5 and 20 cm depth. Description of all measurements and equipment is available in Supplementary Methods I.

### Field data analysis

We tested treatment (factor; irrigation or control) and measurement date (factor) and their interaction for significant effects on field-measured thaw depths, water tables and topsoil volumetric moisture content using mixed-effects models. To account for repeated measurement in a nested setup, we used plot number as a random effect and tested for significance of random intercepts and slopes on a full model with interaction using likelihood ratio tests. The significance of fixed effects was assessed using *F*-tests with Kenward–Rogers approximation of degrees of freedom on nested models^56^. The optimal model structure was determined using backwards selection based on predictor *p* values, Akaike’s Information Criterion, normality and homoscedasticity of residuals and absence of patterns of residuals against random factors and fitted values. We allowed for the transformation of dependent variables and the addition of zero-inflation components to improve residual diagnostics. We performed all statistical analysis in R version 3.5.1^57^ using the lme4 package^58^. An extensive description of the procedures for statistical analysis is available in Supplementary Methods II.

### Modelling study

We used the ATS^33^ version 0.88 to (1) support the field experiment with mechanistic insight and (2) to explore the temperature sensitivity of rainfall effects using several rainfall and temperature scenarios. ATS is a fully coupled surface-subsurface thermal hydrology model, configured for permafrost applications^59^. It couples the surface energy balance and snow dynamics with a subsurface thermal hydrology scheme to represent three-phase freeze and thaw cycles accounting for moisture migration. To run the model, atmospheric data on air temperature, precipitation, incoming shortwave radiation, relative humidity and wind speed is required. Except for incoming shortwave radiation, a time series from 1 January 1966 to 31 July 2020 for all these values was available for Chokurdakh (WMO station code 21649, 30 km northwest of the study site) from the All-Russia Research Institute of Hydrometeorological Information – World Data Centre^54^. Incoming shortwave radiation was retrieved from ERA5 reanalysis data^60^. We set layer definition, parameters and boundary conditions of our one-dimensional model based on field measurements (Supplementary Table 11). We used both direct field measurements from the experimental sites and measurements from earlier studies conducted at representative locations. For a predefined set of thermal and hydrological for which no field data were available, we calibrated parameters for an accurate representation of field-measured thaw depths. We used literature values to define a likely range within which these parameters were calibrated (Supplementary Table 11). We used NSE to quantify the extent to which the modelled depth of the 0 °C isotherm (the depth at which the permafrost table is situated) followed the field-measured thaw depths and set parameters to maximise NSE. In addition, modelled soil moisture and soil temperature at 5 and 20 cm depth were compared visually to logger measurements after calibration. An independent check was performed by comparing model-predicted thaw depths between 2007 and 2017 to field measurement series^32,35,61^ at undisturbed shrub tundra plots situated at approximately 500 m distance from the irrigated sites towards which the model was calibrated. An extensive description of the model calibration procedure and resulting fits with field measurement series is available in Supplementary Methods III and IV.

To assess the sensitivity of modelled thaw dynamics and rainfall effects to selected parameters, we performed a sensitivity analysis on both ALT (or maximum end-of-season depth of the 0 °C isotherm) and rainfall effects on ALT (quantified as the difference in ALT between baseline runs and runs with increased rainfall) using structural increases and decreases in soil thermal-hydrological parameters. As changes in single parameters may not be representative of field-observed variability in soil properties, we modelled thaw depth dynamics across a range of soil stratigraphical (variable peat layer thickness overlying variable mineral soil textural classes) and climatic (various summer temperature and rainfall scenarios) conditions. See Supplementary Methods V for a full description of model sensitivity analysis and analysis of rainfall effects across soil types and temperature scenarios.

We used the calibrated model parametrization to analyse various rainfall scenarios for one summer, followed by 2 years of baseline conditions. We established a baseline scenario with daily precipitation and temperature based on averaged forcing conditions (1979–2018) and several scenarios with varied amounts and timing of rainfall in summer based on frequency-intensity distributions of daily precipitation from the Chokurdakh record^54^. We simulated years with high (70th–80th percentile of total JJA precipitation) and extreme precipitation (95th–100th percentile of total JJA precipitation). To model the role of variability in rainfall within the summer season, high and extreme rainfall scenarios were represented both as uniform increases over the summer season and as variable increases with extreme rainfall events within the season. Furthermore, we added four scenarios with 80 additional mm of rainfall in either June, July, August or September. We complement the rainfall scenarios by adding two temperature scenarios simulating a summer of average temperature (*T*_avg,JJA_ = 7.9 °C), and a very warm summer based on the 95th–100th percentile of mean summer temperature (*T*_avg,JJA_ = 10.6 °C). We compared the ALT and timing of complete freeze-up among the different rainfall scenarios. An elaborate description of the scenario definition is available in Supplementary Methods VI.

## Supplementary information

Supplementary Information

## Data Availability

The data generated in this study have been deposited in the DANS EASY database at 10.17026/dans-xdt-tf9f^62^.

## Code availability

The code generated in this study has been deposited in the DANS EASY database at 10.17026/dans-xdt-tf9f^62^. The code for Amanzi ATS v. 0.88 is available from Coon et al. (2019)^33^ (https://github.com/amanzi/ats).
