# Supplementary material for: Extremely wet summer events enhance permafrost thaw for multiple years in Siberian tundra
Source: Nat Commun. 2022 Mar 23;13:1556. doi: 10.1038/s41467-022-29248-x (PMC8943195; doi:10.1038/s41467-022-29248-x)
Supplement: Supplementary file 1 — Supplementary Information [file 41467_2022_29248_MOESM1_ESM.pdf]

Supplementary Information for "Extremely wet summer events enhance permafrost thaw for multiple years in Siberian tundra"

Rúna Í. Magnússon[1], Alexandra Hamm[2,3], Sergey V. Karsanaev[4], Juul Limpens[1], David Kleijn[1], Andrew Frampton[2,3], Trofim C. Maximov[4] and Monique M. P. D. Heijmans[1].

1. *Plant Ecology and Nature Conservation Group, Wageningen University & Research, Wageningen, The Netherlands*
2. *Department of Physical Geography, Stockholm University, Stockholm, Sweden*
3. *Bolin Centre for Climate Research, Stockholm University, Stockholm, Sweden*
4. *Institute for Biological Problems of the Cryolithozone, Siberian Branch of the Russian Academy of Sciences, Yakutsk, Russia*

Contents

Supplementary Methods I – Fieldwork 4
Supplementary Figure 1) 4
Supplementary Figure 2) 5
Supplementary Table 1) 6
Supplementary Table 2) 6
Supplementary Methods II - Field Data Analysis 8
Supplementary Methods III - Model Definition 9
Supplementary Methods IV - Model Calibration 10
Supplementary Methods V - Sensitivity Analysis & Variability of Rainfall Effect in Different Soil Types 11
Supplementary Table 3) 12
Supplementary Methods VI - Model Scenario Analysis 13
Supplementary Table 4) 14
Supplementary Figure 3) 15
Supplementary Results I - Statistical Analysis of Field Data 16
Supplementary Table 5) 16
Supplementary Table 6) 17
Supplementary Table 7) 18
Supplementary Table 8) 19
Supplementary Table 9) 20
Supplementary Table 10) 21
Supplementary Results II - Soil Temperature & Soil Moisture Logger Data 22
Supplementary Figure 4) 22
Supplementary Figure 5) 23
Supplementary Results III - Experiment Replication using ATS 24
Supplementary Table 11) 24
Supplementary Figure 6) 25
Supplementary Figure 7) 26
Supplementary Figure 8) 28
Supplementary Figure 9) 29
Supplementary Figure 10) 30
Supplementary Figure 11) 30
Supplementary Figure 12) 31
Supplementary Results IV - Sensitivity Analysis 32
Supplementary Figure 13) 32
Supplementary Figure 14) 34
Supplementary Results V - Scenario Results 332
Supplementary Figure 15) 36

Supplementary Figure 16) 37

Supplementary Figure 17) 38

Supplementary Results VI - Miscellaneous 39

Supplementary Figure 18) 337

Supplementary Figure 19) 40

Supplementary Table 12) 41

Supplementary Table 13) 41

References 42

Supplementary Methods I – Fieldwork

Experimental Design

Five sites were selected in dwarf shrub dominated tundra vegetation in a drained thaw lake bed ("alas") close to the Chokurdakh Scientific Tundra Station (Supplementary Figure 1a). Each location was chosen so that a thaw pond or drainage gully with open water was nearby, as a source of irrigation water. In each site, 4 circular plots 5m diameter were set out, with at least 5m between plots (Supplementary Figure 1b). In the centre of each plot a pvc well was installed to monitor the water table (WT) above the permafrost. Prior to the irrigation experiment we measured thaw depth (TD) and soil volumetric moisture content (SM) in nine locations per plot; eight points along the perimeter of the plot at 1m distance from the plot edge, and one in the centre (1c). Cover of the main vegetation species and variation in microtopography was assessed visually. Plots were assigned to pairs based on similarity in initial TD, WT, SM, vegetation composition and microtopography. Plots from pairs were randomly assigned to irrigation and control, although in a few cases the length of the hoses of the irrigation system dictated the subdivision. Statistical analysis confirmed similarity in TD, WT, SM (fig. 1 main text) and microtopography (Supplementary Figure 19) at the start of the experiment.

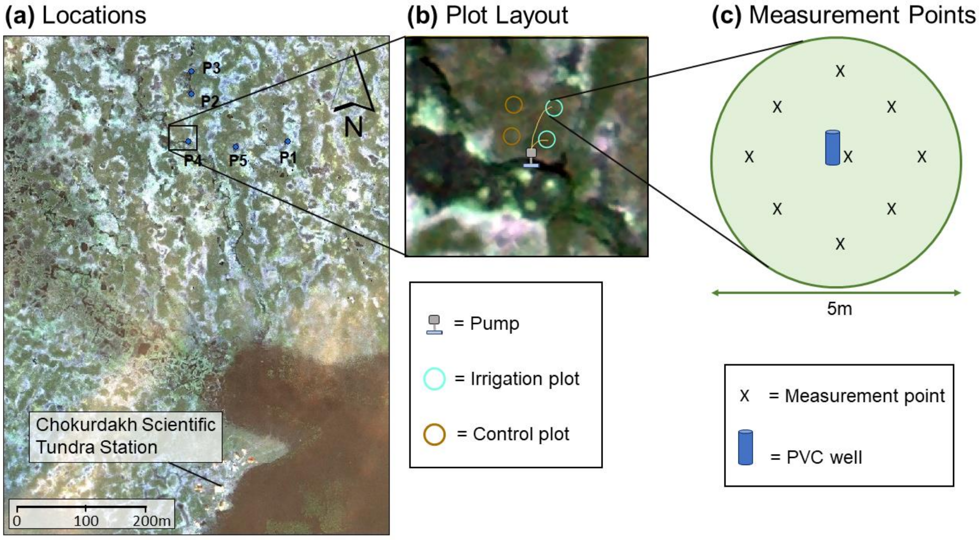

Supplementary Figure 1) a) *5 selected sites.* ***b)*** *Each site consists of two control plots and two irrigation plots, which are supplied with local surface water by a motor pump.* ***c)*** *In each plot, nine approximate measurement points were set out and one pvc well was installed. Map image in* ***a*** *and* ***b****: WorldView-2 © 2019 Maxar Technologies*

Irrigation Protocol

The plots assigned to the irrigation treatment received 100mm of irrigation over the period of July 6th to August 2nd 2018. The irrigation water was supplied from local water bodies (ponds, gullies) using a filter tube, motor pump and sprinkler set-up (Supplementary Figure 2). The temperature of rainwater is generally expected to be the same as that of ambient air[1,2]. Prior to irrigation, the temperature of the water at approximately 10cm depth was measured using a handheld thermometer (HI 9063 K-couple Thermometer, Hanna Instruments, Padua, Italy). With water temperatures between 9.1 and 9.3°C and an average July ambient air temperature of 9.8°C[3], the temperature of irrigation water did

not exceed average ambient air temperature during the experiment. Samples were taken from each water body and from rainwater collected on the site. Water sample analysis confirmed that surface water chemical composition did not deviate substantially from that of rainwater (Supplementary Table 2). The filter tube had .63mm slits and was attached to a floating tube and positioned at 10cm below the water surface. Sprinklers were installed on ± 0.5m high statifs to reach above the canopy and to achieve a 2.5m irrigation radius. We used a two-stroke motor pump that supplied two sprinklers per site simultaneously via 10m hoses with an approximate irrigation rate of 25mm hr^{-1}.

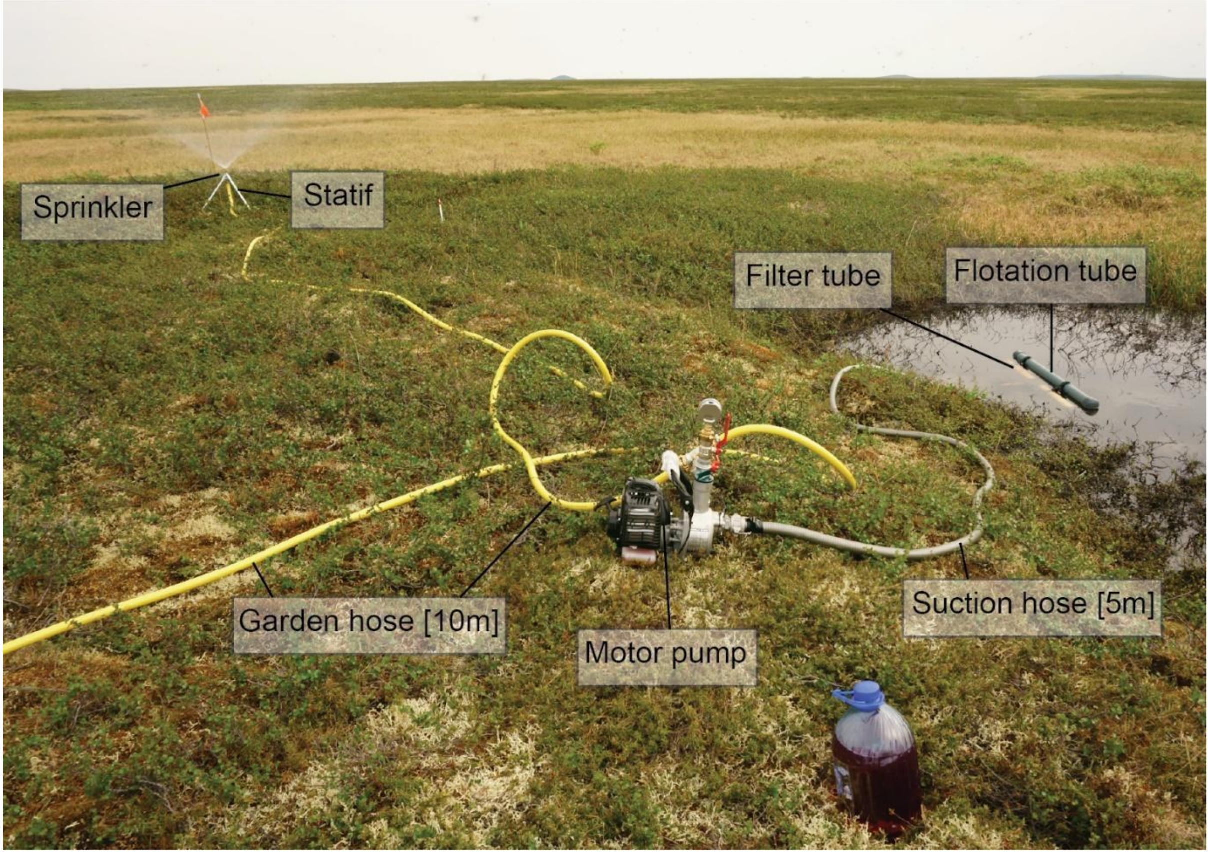

Supplementary Figure 2) *Technical setup used for irrigation.*

Irrigation was supplied in portions of 10 or 15mm (± 20-40 minutes session) on an approximately biweekly basis (Supplementary Table 1). The amount of irrigation was monitored using rain gauges. Irrigation was supplied on relatively windless days to assure uniform distribution of irrigation water over plots. Due to technical difficulties, several plots received more or less irrigation during particular sessions, but each plot received between 97 and 103 mm of irrigation in total over the course of the experiment.

Supplementary Table 1) *Timing and amounts of irrigation [mm] for all 10 irrigated plots. P1 - P5 refer to site locations (Supplementary Figure 1a), and I1-I2 refer to the two irrigated plots per site (Supplementary Figure 1b).*

plot	06-Jul	10-Jul	12-Jul	16-Jul	21-Jul	23-Jul	28-Jul	29-Jul	02-Aug	TOTAL
P1I1	10	9.5	10	10	15	15	15	0	12.5	97
P1I2	10	9.5	10	10	15	15	15	0	12.5	97
P2I1	10	12	10	10	15	15	15	0	11	98
P2I2	10	12	10	10	15	15	15	0	16	103
P3I1	10	0	20	10	15	15	15	0	15.5	100.5
P3I2	10	0	20	10	15	15	15	0	15.5	100.5
P4I1	10	10.5	10	10	15	15	15	0	14	99.5
P4I2	10	10.5	10	10	15	15	15	0	14	99.5
P5I1	10	10	10	10	15	15	8.5	3	16	97.5
P5I2	10	10	10	10	15	15	8.5	3	16	97.5

Supplementary Table 2) *Concentration of main anions and cations in surface water used for irrigation compared to on-site rainwater. Surface water used for irrigation was sampled at each site (P1-P5). A rainwater sample was collected with a rain gauge over the course of several days in early August 2018. N.D.= not detected*

	K mg/L	Na mg/L	Mg mg/L	Ca mg/L	N-NO3 mg/L	N-NH4 mg/L	P-PO4 mg/L	SO4 mg/L	Cl mg/L
RAIN	2.48	N.D.	0.56	3.97	N.D.	1.03	N.D.	41.66	2.32
P1	2.05	N.D.	1.52	1.94	N.D.	1.3	N.D.	8.74	1.85
P2	3.92	N.D.	2.02	2.21	N.D.	1.85	N.D.	4.08	2.63
P3	3.42	N.D.	1.52	2.07	N.D.	0.25	N.D.	1.84	2.94
P4	2.22	N.D.	1.86	1.46	N.D.	0.84	N.D.	5.57	2.32
P5	6.96	N.D.	1.34	1.45	N.D.	0.83	N.D.	5.51	2.01

Field Measurements

Field measurements of thaw depth, water tables and volumetric moisture content of the topsoil that were carried out prior to the start of the experiment (July 2nd) were repeated twice (July 25th and August 4th) in 2018. Both of these measurement rounds were timed 2 days after the most recent irrigation session. Measurements were repeated three times during the summer of 2019 (June 26th, July 22nd and August 7th) and only twice during the summer of 2020 (July 22nd and August 12th, due to travel restrictions). Thaw depth was measured in 9 points per plot (Supplementary Figure 1c) as the distance between the top of the permafrost and the soil/moss surface using a blunt-tipped metal rod. Water tables were measured as the height of standing water above the frozen soil by lowering down a ruler with centimeter scale into the PVC wells in the centre of each plot and noted as “0” when absent. Volumetric moisture content of the topsoil was measured in the top 5cm of the soil/moss surface using a Thetaprobe ML2X (Delta-T Devices, Burwell, UK) calibrated for local organic soils in 9 locations per plot (Supplementary Figure 1c).

Microtopography was assessed using an optical leveller (Kompensator-Nivellier NI 025, VEB Carl Zeiss, Jena, Germany) that was installed in each site to assess difference in relative elevation among plots within the same site. Over each plot, transects were laid out in north-south and east-west orientation, extending 2 meters outside the plot on both sides. Relative elevation was measured at 0.5m intervals.

At the onset of the irrigation experiment, eight plots (four control, four irrigated) were equipped with iButton DS1921G-F5# (Maxim Integrated Products, Sunnyvale, California, USA) temperature sensors and S-SMC-005 soil moisture sensors connected to a HOBO H21-USB microstation (both Onset Computer Corporation, Bourne, Massachusetts, USA) at depths of 5 and 20 cm. Soil temperature was recorded every three hours and soil volumetric soil moisture was recorded every hour. From these measurement series, daily averages were calculated for soil temperature and volumetric moisture. Due to technical issues the record for the iButton measurements shows a gap between September and November 2018.

Supplementary Methods II - Field Data Analysis

We used the lme4 package[4] in R version 3.5.1[5] to fit (generalized) linear mixed effects models (GLMMs) to explain observed dynamics in abiotic variables measured in the plots. Treatment (factor; irrigation or control), date of measurement (factor) and their interactions were selected as fixed factors and tested for significant effects on abiotic conditions. To account for repeated measurement in a nested set-up, we used plot number (n = 20) as a random effect and tested for significant random intercept and significant random slopes for treatment and date using likelihood ratio tests (LRTs) on nested models with a backward selection procedure. Random structures were tested on full models (all fixed effects incl. interactions) assuming an unstructured variance-covariance structure and considering both correlated and uncorrelated random slopes and intercepts. The optimal random structure was selected based on LRTs, model convergence and Akaike's Information Criterion (AIC). Using the selected random structure, significance of fixed effects was assessed using F-tests with Kenward-Rogers approximation of degrees of freedom on nested models using the pbkrtest package[6] in a backwards selection procedure. The optimal model structure was selected based on predictor p-values, AIC, normality and homoscedasticity of residuals and absence of patterns of residuals against random factors and fitted values. Specific additional adjustments were made to account for the distribution of dependent variables and improve residual diagnostics:

- Thaw Depth - was square root transformed to improve residual distribution.
- Water Table - was frequently absent throughout all measurement dates, but too few observations (one per plot) were available to add a zero-inflation component. As a solution, the water table was assessed using logistic GLMM (presence vs. absence of a water table), following the same procedure as described above. As F-tests are not available for GLMMs[6], LRTs were used to assess the significance of fixed effects.
- Soil Moisture - was square-root transformed to improve residual distribution. Due to frequent absence of moisture in the topsoil in 2019, a zero-inflation component dependent on the date of measurement was added to the model formula for SM using the glmmTMB package[7]. Resulting GLMMs were evaluated for residual distribution, overdispersion and zero-inflation using the DHARMa R package[8].

For all three selected models, the significance of the difference between irrigated and control plots was assessed per individual measurement date using Tukey contrasts with Bonferroni correction for multiple testing.

Measurements of relative elevation were used to check for differences between the microtopographical position of control and irrigated sites at the start of the experiment, and to check for potential influence of microtopogtaphy on thaw depths. The mean elevation of all outside-plot measurements within a site was subtracted from each within-plot measurement to obtain standardized relative elevations. Differences in microtopographical position between control and irrigated sites were tested using a mixed linear effect model with a random intercept for each unique plot. F-tests with Kenward-Rogers approximation of degrees of freedom[6] were used to test for the significance of treatment. Relations between the average microtopographical position of plots within sites and plot-averaged end-of-summer thaw depths and water table in plot centres in 2018 were assessed using a linear model.

Supplementary Methods III - Model Definition

The Advanced Terrestrial Simulator (ATS) couples the surface energy balance with subsurface energy and mass fluxes accounting for three-phase freeze and thaw processes. For details on the model definition and specific permafrost configuration, refer to Atchley et al. (2016)[9] and Painter et al. (2016)[10].

To initiate the model, a three-step spin-up procedure was employed, consistent with methodological developments for Arctic hydrogeological modelling[9-11]. Our model is defined as a 1D column, expanding 1m in x and y direction and 20m in z direction. Individual cells have a thickness of 1cm (up to 20cm) to 2cm (20cm to 50cm depth) near the surface and progressively higher thickness below to accurately represent freeze- and thaw processes and increase with depth. To represent an average subsurface stratigraphy of the landscape[12], our column consists of a 5cm thick moss layer on the top, underlain by 15cm of organic material. Below the organic material, the rest of the column is considered to be mineral soil. A water table is established by defining bottom and surface pressure for non-freezing conditions (step 1). The column is then frozen from the base by assigning a bottom temperature boundary condition corresponding to site conditions. This freezing from below also accounts for volume expansion from liquid to frozen water, allowing unfrozen water to be expelled through the top of the model boundary (step 2). In the last step, the full surface energy balance is included - accounting for surface-subsurface interactions of heat and water flow - and is run until an annual steady-state is obtained where year-to-year changes in temperature and moisture are minimal (step 3). Typically, several decades of annual iterations are needed to attain this periodic convergence. In our case, we ran the model with daily values for the Chokurdakh weather station (WMO station code 21946) 30km northwest of the study site, obtained from the All-Russia Research Institute of Hydrometeorological Information - World Data Centre[3] complemented by ERA5 radiation data from 1979 to 2019[13]. The transient model run to reproduce the field experiment is then initiated with the final model state corresponding to January 1st 2009 from the end of the spin up in step 3. Daily weather data from 2009 to 2019 is used to inform the model for the transient run. For the results, only the years 2018 and 2019 were used. The period from 2009 to 2017 is used again as a spin up in each transient simulation as initial conditions might alter the results in the first years. This way we ensure that the system is in steady state again when producing the final results.

Supplementary Methods IV - Model Calibration

Model parameters for the soil properties of the column need to be assigned. These include bulk density, porosity, permeability, thermal conductivity (κ, for dry, unfrozen and saturated, unfrozen), soil water retention parameters (van Genuchten parameters m and α), surface roughness length and bottom boundary temperature. Each parameter (except for surface roughness and bottom boundary temperature) is defined for each soil layer separately. Bulk density and density derived porosity is based on field measurements[12]. The bottom boundary temperature is derived from GTN-P database for a borehole in the study area (141°49 E, 70.83°N, "Kytalik_GI-01_7m")[14]. Surface roughness length was determined from fieldwork observations of vegetation height and literature values[15]. An overview over these parameters is given in Supplementary Table 11. Parameters without comprehensive site-specific measurements include thermal conductivity, van Genuchten parameters and permeability for all three layers. Literature values are used for permeability for moss, peat and mineral layers obtained from Jan et al. (2020)[11]. We performed a systematic parameter combination assessment first for the water retention parameters and afterwards for the thermal conductivity parameters. A range of values was defined based on literature-averaged values and systematic increase and decrease of 25% or 50%, resulting in three different values for each layer to be tested. This yielded 729 different parameter combinations to evaluate. Parameter ranges and selected values used can be found in Supplementary Table 11. In a first step, all 729 runs covered the entire spin up period from 1979 to 2018 to ensure steady state by 2009, which serves as the starting point for transient simulations. In 2018, daily values for temperature in 5 and 20cm, soil moisture in 5 and 20cm and depth of the 0°C isotherm were written to an output file, which is fed into the evaluation against field data. Modelled daily values were compared to spatially average field measured thaw depth (Figure 1c, Main Text), soil moisture and temperature (Supplementary Figure 4-5) and parameters were selected by maximizing the Nash-Sutcliffe Efficiency (NSE) of the modelled thaw depth data against the measured data. Based on the best results for the water retention evaluators m and α, thermal parameters (κ) were changed in a similar manner, but runs were initiated in 2009 instead of 1979. The same output as in the previous step was compared again against field data and the best match for thaw depth was determined to be the best fit. We chose thaw depth as the calibration target as it represents the combined effect of thermal- and hydrological parameters and as the evaluation has shown that local variability in field-measured soil moisture is high (Supplementary Figure 4). As a result, it was not possible to perfectly match all the different field observations simultaneously. As a final test, predicted thaw depths for 2007 – 2018 were compared to a historical measurement series (2007-2017) of thaw depths from 5 undisturbed shrub tundra sites situated at approximately 500m distance from the irrigated sites[16-18].

Supplementary Methods V - Sensitivity Analysis & Variability of Rainfall Effect in Different Soil Types

Parameter Sensitivity Analysis

A model sensitivity analysis was performed on the calibrated parameters (van Genuchten m, van Genuchten α, dry and saturated thermal conductivity of each soil layer), as well as site specific parameters related to stratigraphy and microtopography (thickness of the peat layer, maximum ponded depth), see Supplementary Table 11. Sensitivity analyses for calibrated parameters was performed by structurally lowering or increasing each parameter value by 5% and 10% and evaluating resulting end-of-season active layer thicknesses compared to those obtained using the calibrated parameter set from Supplementary Table 11. For peat layer thickness and maximum ponded depth, stratigraphical and microtopographical data were available from Magnússon et al., (2020)[19] to inform likely ranges of spatial heterogeneity. For these parameters, ranges of 8cm – 10cm – 15cm – 20cm – 30cm – 26cm (peat layer thickness) and -10cm - -5cm – 0 cm – 5cm – 10cm (maximum ponded depth) were used to assess model sensitivity.

For the sensitivity analysis, several summer precipitation scenarios were used, preceded by spin-up runs described in Supplementary Methods III.

1) A baseline precipitation scenario with an annual series of daily temperature and precipitation values representing average summer precipitation dynamics and average daily temperatures for the period 1944-2019 (see Supplementary Methods VI, Supplementary Figure 3a), with a total of 77mm precipitation in June-August.
2) A summer with averaged temperatures and a sustained high precipitation (see Supplementary Methods VI, Supplementary Figure 3b), with a total of 104mm precipitation in June-August, distributed uniformly over days in June-August.
3) A summer with averaged temperatures and a precipitation extreme in July (see Supplementary Methods VI, Supplementary Figure 3f), with a total of 157mm precipitation in June-August, represented by baseline precipitation + 80mm in July.

For each parameter, soil layer and scenario, an elasticity index (EI) was calculated[20], representing the %-change in ALT relative to the %-change in the parameter value. EI was also calculated for the rainfall effect, representing the %-change in magnitude (relative to baseline precipitation) of the rainfall effect relative to the %-change in parameter values. If ALT or rainfall effects responded very nonlinearly to parameter changes or could not be calculated due to lack of model convergence for adjusted parameters, no EI was calculated.

Rainfall effects in different soil types

Apart from changing parameter values individually, we extended the sensitivity analysis over various mineral soil types, represented by the main USDA soil textural classes[21]. The rationale here was to allow for simultaneous changes in thermal and hydrological behaviour of soils as a result of variability in texture and stratigraphy, rather than changing a single parameter at a time. We ran different rainfall scenarios (see Supplementary Methods VI) for 12 mineral texture classes[21] and corresponding soil hydrological[22] and thermal[23] parameters, see Supplementary Table 3. Parameters of the moss and peat layer were kept at calibrated values. The resulting ALTs and rainfall effects sizes per soil textural class were generated for different peat layer thickness (15cm and 25cm) and temperature scenarios

(for an average summer and a very warm summer, see Supplementary Methods VI). All other parameters and input data were kept at calibrated values (Supplementary Table 11).

Supplementary Table 3) USDA mineral soil textural classes and corresponding hydrological and thermal parameters used for extended sensitivity analysis

Soil Type	Bulk Density	Porosity	^{10}log(vG alpha)	^{10}log(vG n)	Residual Water Content	Dry Thermal Capacity	Saturated Thermal Capacity	Dry Thermal Conductivity	Saturated Thermal Conductivity	%-Sand**	%-Silt**	%-Clay**
Clay	1.35	0.459	-1.825	0.098	0.098	1.55	2.4	0.64	1.9	0.2	0.15	0.65
Clay Loam	1.45	0.442	-1.801	0.151	0.079	1.52*	2.43*	0.541*	2.32*	0.3	0.3	0.4
Loam	1.5	0.399	-1.954	0.168	0.061	1.51*	2.44*	0.508*	2.4	0.4	0.4	0.2
Loamy Sand	1.6	0.39	-1.459	0.242	0.049	1.472*	2.478*	0.439*	2.551*	0.78	0.15	0.07
Sand	1.65	0.375	-1.453	0.502	0.053	1.45	2.5	0.4	2.6	0.9	0.07	0.03
Sandy Clay	1.4	0.385	-1.476	0.082	0.117	1.5*	2.45*	0.511*	2.32*	0.5	0.1	0.4
Sandy Clay Loam	1.5	0.384	-1.676	0.124	0.063	1.489*	2.461*	0.482*	2.418*	0.61	0.13	0.26
Sand Loam	1.55	0.387	-1.574	0.161	0.039	1.485*	2.465*	0.462*	2.53*	0.65	0.25	0.1
Silt	1.5	0.489	-2.182	0.225	0.05	1.55	2.4	0.55	2.6	0.09	0.85	0.06
Silty Clay	1.45	0.481	-1.79	0.121	0.111	1.545*	2.405*	0.586*	2.264*	0.05	0.47	0.48
Silty Clay Loam	1.5	0.482	-2.076	0.182	0.09	1.54*	2.41*	0.566*	2.362*	0.1	0.56	0.34
Silt Loam	1.5	0.439	-2.296	0.221	0.065	1.53*	2.42*	0.534*	2.495*	0.2	0.65	0.15
Unit	[kg m^{-3}]	[cm^3 cm^{-3}]	[cm^{-1}]	[-]	[cm^3 cm^{-3}]	[J m^{-3} K^{-1}]	[J m^{-3} K^{-1}]	[W m^{-1} K^{-1}]	[W m^{-1} K^{-1}]	[g g^{-1}]	[g g^{-1}]	[g g^{-1}]
Reference	[21]	[22]	[22]	[22]	[22]	[23]	[23]	[23]	[23]	[21]	[21]	[21]

*) *Thermal properties interpolated based on percentages of sand, silt and clay endmembers.*

**) *Estimates of average fractions per class, derived from USDA Soil Texture Triangle*[21]

Supplementary Methods VI - Model Scenario Analysis

Scenario Definition

Precipitation scenarios were established based on the Chokurdakh station climate data[3]. For the scenario analysis, a distinction was made between scenarios with extreme precipitation events and scenarios with a uniform increase in precipitation over the entire summer season (June – August).

For the scenarios with extreme precipitation events, a "baseline", "high" and "extreme" summer precipitation scenario were generated based on frequency-intensity distributions of daily precipitation (1944-2019) in years with average, wet and extremely wet summers. Regular years were defined as years within the 40th - 50th percentile of total summer (JJA) precipitation. High summer precipitation years were defined as years within the 70th - 80th percentile of total summer (JJA) precipitation. Extreme summer precipitation years were defined as the wettest 5% of summer (95th-100th percentile of total summer precipitation). Using the frequency-intensity distribution of rainfall in the summer periods selected sets of years (4 < n < 8), artificial distributions were generated for each scenario for one year, yielding total summer precipitation of 77mm, 104mm and 178mm. Across these three scenarios, timing of precipitation events was kept constant by putting the highest to lowest daily precipitation in the same order across scenarios, where the order was derived from the long-term averaged daily precipitation.

In addition, to better constrain the role of timing of precipitation extremes, 4 scenarios were generated where 80mm of additional precipitation was added to the "baseline" scenario in a particular month divided over 4 equally spread days per month.

To compare the effect of extreme precipitation events following observed frequency-intensity distributions to scenarios with uniformly distributed increases in precipitation, two additional scenarios were created ("high uniform" and "extreme uniform"). For these scenarios, the additional precipitation for the high and extreme scenario (104mm – 77mm = 27mm and 178mm – 77mm = 101mm) were added to the baseline scenario, distributed equally over all days in June-August that did not already show precipitation in the baseline scenario.

Each scenario was followed by 2 years of "baseline" precipitation to allow for the observation of carry-over effects. The scenarios are summarized in Supplementary Table 4.

Temperature Scenarios

The resulting 9 scenarios were run under average summer temperature conditions and extremely warm summer temperature conditions, based on average daily summer temperature in regular summers (40th - 50th percentile of mean summer temperature, n = 7) and the warmest 5% of summers (95th- 100th percentile of mean summer temperature, n = 4). All non-summer conditions were set to long term daily averages from the entire climate record. Other inputs (incoming shortwave radiation, relative humidity and wind speed) were set to daily averages over the forcing period (see Supplementary Methods III).

Supplementary Table 4) Summer precipitation scenario definitions

Scenario	Representation	Precipitation distribution	Total JJA precipitation
Baseline	Summers with average rainfall amounts: 45th – 55th percentile of total summer precipitation	Frequency-Intensity distribution of summers within 45th – 55th percentile of total summer precipitation	**77mm**
High – Variable	Summers with high rainfall amounts: 70th – 80th percentile of total summer precipitation	Frequency-Intensity distribution of summers within 70th – 80th percentile of total summer precipitation	**104mm**
High – Uniform	Summers with high rainfall amounts: 70th – 80th percentile of total summer precipitation	Baseline + uniform increase over June-August	**104mm**
Extreme – Variable	Summers with extreme rainfall: 95th – 100th percentile of total summer precipitation	Frequency-Intensity distribution of summers within 70th – 80th percentile of total summer precipitation	**178mm**
Extreme – Uniform	Summers with extreme rainfall: 95th – 100th percentile of total summer precipitation	Baseline + uniform increase over June-August	**178mm**
June Increase	Summers with 4 days of extreme rainfall [20mm each] in June, otherwise baseline.	Baseline + 4 times 20mm in June	**157mm**
July Increase	Summers with 4 days of extreme rainfall [20mm each] in July, otherwise baseline.	Baseline + 4 times 20mm in July	**157mm**
August Increase	Summers with 4 days of extreme rainfall [20mm each] in August, otherwise baseline.	Baseline + 4 times 20mm in August	**157mm**
September Increase	Summers with 4 days of extreme rainfall [20mm each] in September, otherwise baseline.	Baseline + 4 times 20mm in September	**77mm (JJA) + 103mm (Sept)**

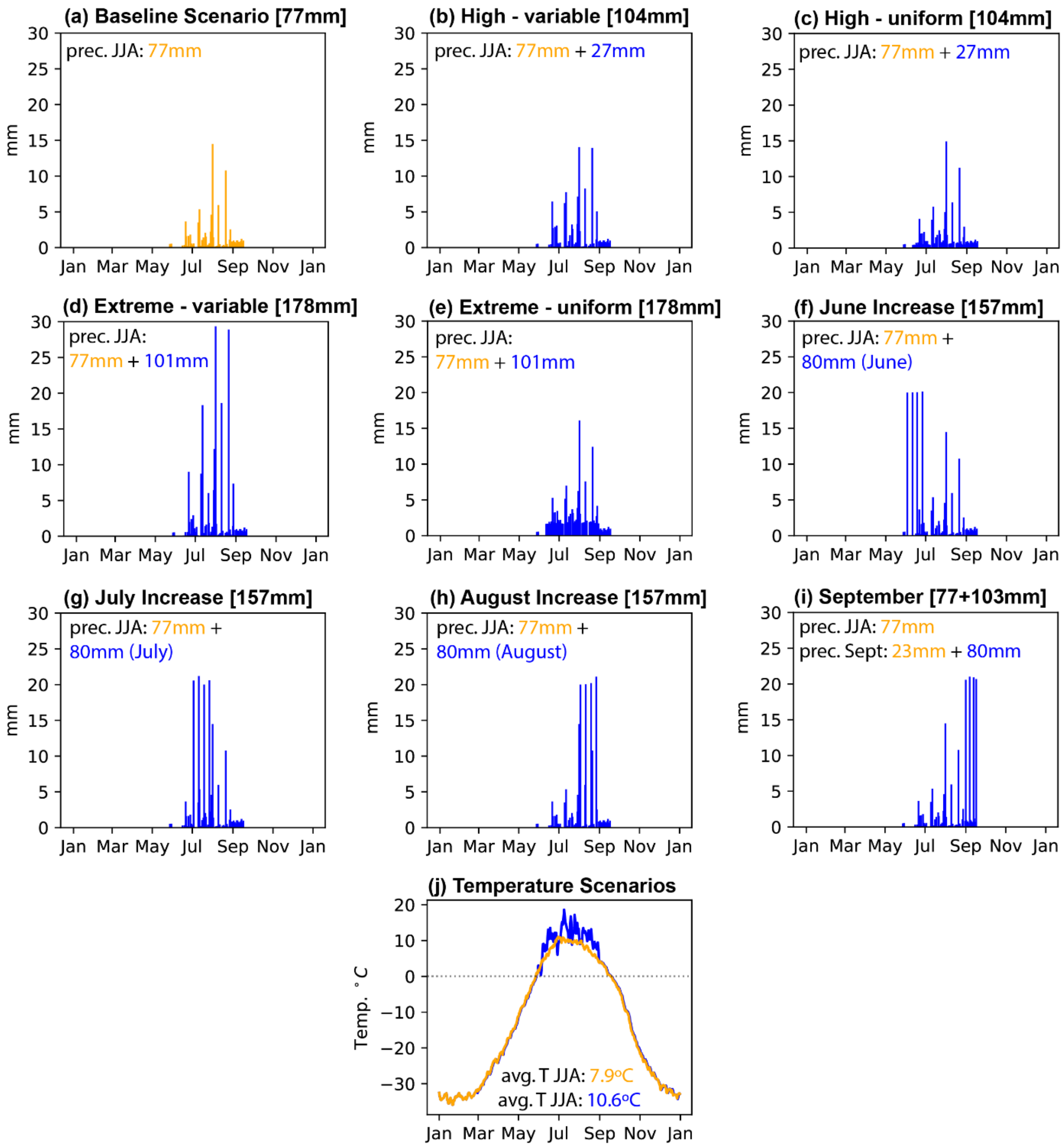

Supplementary Figure 3) *Daily precipitation and temperature scenarios (see Supplementary Table 4) used for the scenario study.* ***a)*** *Baseline scenario (77mm).* ***b)*** *High precipitation scenario with variable increase (104mm).* ***c)*** *High precipitation scenario with uniform increase (104mm).* ***d)*** *Extreme precipitation scenario with variable increase (178mm).* ***e)*** *Extreme precipitation scenario with uniform increase (178mm).* ***f)*** *June increase (157mm).* ***g)*** *July increase (157mm).* ***h)*** *August increase (157mm).* ***i)*** *September increase (157mm June-August + 80mm September).* ***j)*** *Temperature scenarios: orange line indicates average summer (June-August) temperature (7.9 °C), blue line indicates very warm summer (June-August) temperature (10.6 °C).*

Supplementary Results I - Statistical Analysis of Fieldwork Data

Supplementary Table 5) Candidate LMMs for thaw depth. Selected model indicated in bold.

Predictor	Model	AIC	BIC	LL	F-test on nested models		
					F	df	p value
Treatment	sqrt(ALT) ~ moment + treatment + (1 | plot)	2429.827	2487.823	-1203.913	13.29	18	$1.85e^{-3}$
	sqrt(ALT) ~ moment + (1 | plot)	2435.714	2488.437	-1207.857			
Date	sqrt(ALT) ~ moment + treatment + (1 | plot)	2429.827	2487.823	-1203.913	181.05	1413	$< 1e^{-15}$
	sqrt(ALT) ~ treatment + (1 | plot)	3294.988	3316.078	-1643.494			
*Treatment*Date*	**sqrt(ALT) ~ moment * treatment + (1 | plot)**	2404.705	2499.648	-1184.372	8.66	1406	$1.97e^{-10}$
	sqrt(ALT) ~ moment + treatment + (1 | plot)	2429.827	2487.823	-1203.913			
					Likelihood Ratio Test		
					X^2	df	p
Random intercept for Plot	**sqrt(ALT) ~ moment * treatment + (1 | plot)**	2404.705	2499.648	-1184.372	182.13	1	$< 1e^{-15}$
	sqrt(ALT) ~ moment * treatment	2527.386	2617.017	-1246.693			

Supplementary Table 6) Tukey Contrasts for thaw depth based on selected model.

Date	Treatment	Estimated Marginal Mean ± SE	Estimated Difference (I - C)	Standard Error of Difference	df	Z ratio	P value
02-07-2018	Irrigation	12.3 ± 0.65	0.513	0.915	38.4	-0.560	0.579
	Control	11.8 ± 0.64					
25-07-2018	Irrigation	23.0 ± 0.90	5.017	1.195	38.4	-4.197	0.0002
	Control	18.0 ± 0.79					
04-08-2018	Irrigation	26.3 ± 0.96	6.305	1.270	38.4	-4.965	< 0.0001
	Control	20.0 ± 0.83					
26-06-2019	Irrigation	18.9 ± 0.81	0.865	1.136	38.4	-0.761	0.452
	Control	18.1 ± 0.79					
22-07-2019	Irrigation	27.1 ± 0.97	2.445	1.343	38.4	-1.821	0.077
	Control	24.6 ± 0.93					
07-08-2019	Irrigation	29.1 ± 1.01	4.315	1.371	38.4	-3.148	0.003
	Control	24.8 ± 0.93					
22-07-2020	Irrigation	20.9 ± 0.85	4.249	1.143	38.4	-3.716	0.0006
	Control	16.6 ± 0.76					
12-08-2020	Irrigation	21.6 ± 0.87	5.607	1.144	38.4	-4.900	<0.0001
	Control	16.0 ± 0.75					

Supplementary Table 7) Candidate GLMMs (binomial distribution) for water table. Selected model indicated in bold.

Predictor	Model	AIC	BIC	LL	LRT-test on nested models		
					X2	df	p value
Treatment	WT ~ moment + treatment + (1 | plot)	115.18	167.46	-40.592	26.588	1	< 1e^{-6}
	WT ~ moment + (1 | plot)	144.86	172.53	-63.429			
Date	WT ~ moment + treatment + (1 | plot)	115.18	167.46	-40.592	82.793	7	< 1e^{-14}
	WT ~ treatment + (1 | plot)	189.06	198.29	-91.531			
*Treat*Date*	**WT ~ moment * treatment + (1 | plot)**	120.27	151.02	-50.135	19.087	7	0.0079
	WTP ~ moment _ treatment + (1 | plot)	115.18	167.46	-40.592			
(1 | Plot) (random intercept)	**WT ~ moment * treatment + (1 | plot)**	120.27	151.02	-50.135	0.8736	1	0.35*
	WT ~ moment * treatment	114.06	163.26	-41.028			

**)* *Random intercept was kept to account for repeated measures*

Supplementary Table 8) Tukey Contrasts for water table based on selected model. No degrees of freedom are estimated for contrasts derived from GLMMs.

Date	Treatment	Probability WTP > 0	Estimated Difference (I/C)	Standard Error of Difference	- df	Z ratio	P value
02-07-2018	Irrigation	$2.843e^{-9} \pm 1.604e^{-5}$	$2.0371e^{-9}$	$1.814e^{-5}$	-	$-1.123e^{-4}$	1.000
	Control	$8.067e^{-10} \pm 8.485e^{-6}$					
25-07-2018	Irrigation	$1.000 \pm 1.651e^{-5}$	NA	NA	-	NA	NA (P.S.)*
	Control	$2.220e^{-16} \pm 4.594e^{-9}$					
04-08-2018	Irrigation	$1.000 \pm 1.295e^{-5}$	NA	NA	-	NA	NA (P.S.)*
	Control	$2.220e^{-16} \pm 4.712e^{-9}$					
26-06-2019	Irrigation	$7.840e^{-2} \pm 8.571e^{-2}$	$-7.840e^{-2}$	$8.571e^{-2}$	-	$-9.148e^{-1}$	0.3603
	Control	$1.482e^{-10} \pm 3.637e^{-6}$					
22-07-2019	Irrigation	$7.285e^{-1} \pm 1.512e^{-1}$	$-6.515e^{-1}$	$1.730e^{-1}$	-	-3.766	0.0002
	Control	$7.707e^{-2} \pm 8.404e^{-2}$					
07-08-2019	Irrigation	$3.843e^{-1} \pm 1.700e^{-1}$	$-3.073e^{-1}$	$1.896e^{-1}$	-	-1.621	0.1051
	Control	$7.707e^{-2} \pm 8.404e^{-2}$					
22-07-2020	Irrigation	$9.210e^{-1} \pm 8.521e^{-2}$	$-8.800e^{-2}$	$1.502e^{-1}$	-	-0.586	0.5579
	Control	$8.330e^{-1} \pm 1.237e^{-1}$					
12-08-2020	Irrigation	$9.210e^{-1} \pm 8.521e^{-2}$	$-4.216e^{-1}$	$1.956e^{-1}$	-	-2.155	0.0311
	Control	$4.994e^{-1} \pm 1.761e^{-1}$					

Supplementary Table 9) Candidate GLMMs (gaussian distribution) for soil moisture. Selected model indicated in bold.

Predictor	Model	AIC	BIC	LL	LRT-test on nested models		
					X2	df	p value
Treatment	sqrt(SM) ~ moment + treatment + (1 | plot) + ziformula ~ moment	4821.5	4921.7	-2391.7	11.563	1	0.0007
	sqrt(SM) ~ moment + (1 | plot) + ziformula ~ moment	4831.1	4926.0	-2397.5			
Date	sqrt(SM) ~ moment + treatment + (1 | plot) + ziformula ~ moment	4821.5	4921.7	-2391.7	817.18	7	$<1e^{-15}$
	sqrt(ALT) ~ treatment + (1 | plot) + ziformula ~ moment	4821.5	4921.7	-2391.7			
*Treatment * Date*	**sqrt(SM) ~ moment * treatment + (1 | plot) + ziformula ~ moment**	4629.6	4766.6	-2288.8	205.93	7	$<1e^{-15}$
	sqrt(SM) ~ moment + treatment + (1 | plot) + ziformula ~ moment	4821.5	4921.7	-2391.7			
Zero Inflation	**sqrt(SM) ~ moment * treatment + (1 | plot) + ziformula ~ moment**	4629.6	4766.6	-2288.8	87.723	8	$<1e^{-14}$
	sqrt(SM) ~ moment * treatment + (1 | plot)	4701.3	4796.2	-2332.6			
Random Intercept for Plot	**sqrt(SM) ~ moment * treatment + (1 | plot) + ziformula ~ moment**	4629.6	4766.6	-2288.8	7.1435	1	0.0075
	sqrt(SM) ~ moment * treatment + ziformula ~ moment	4634.7	4766.5	-2292.3			

Supplementary Table 10) Tukey Contrasts for soil moisture based on selected model.

Date	Treatment	Estimated Marginal Mean ± SE	Estimated Difference (I-C)	SE of Difference	df	Z ratio	P value
02-07-2018	Irrigation	20.44 ± 1.221	0.447	1.736	38.4	-0.257	0.7982
	Control	19.99 ± 1.235					
25-07-2018	Irrigation	29.39 ± 1.480	13.762	1.832	38.4	-7.511	<0.0001
	Control	15.63 ± 1.080					
04-08-2018	Irrigation	24.54 ± 1.353	16.675	1.555	38.4	-10.762	<0.0001
	Control	7.87 ± 0.766					
26-06-2019	Irrigation	3.797 ± 0.636	2.558	0.725	38.4	-3.531	0.0009
	Control	1.239 ± 0.385					
22-07-2019	Irrigation	5.467 ± 0.638	-0.472	0.922	38.4	0.511	0.6119
	Control	5.938 ± 0.665					
07-08-2019	Irrigation	2.278 ± 0.574	-1.001	0.857	38.4	1.168	0.2494
	Control	3.278 ± 0.666					
22-07-2020	Irrigation	16.585 ± 1.112	-8.470	1.762	38.4	4.807	<0.0001
	Control	25.055 ± 1.367					
12-08-2020	Irrigation	23.608 ± 1.327	-3.001	1.935	38.4	1.551	0.1290
	Control	26.608 ± 1.409					

Supplementary Results II - Soil Temperature & Soil Moisture Logger Data

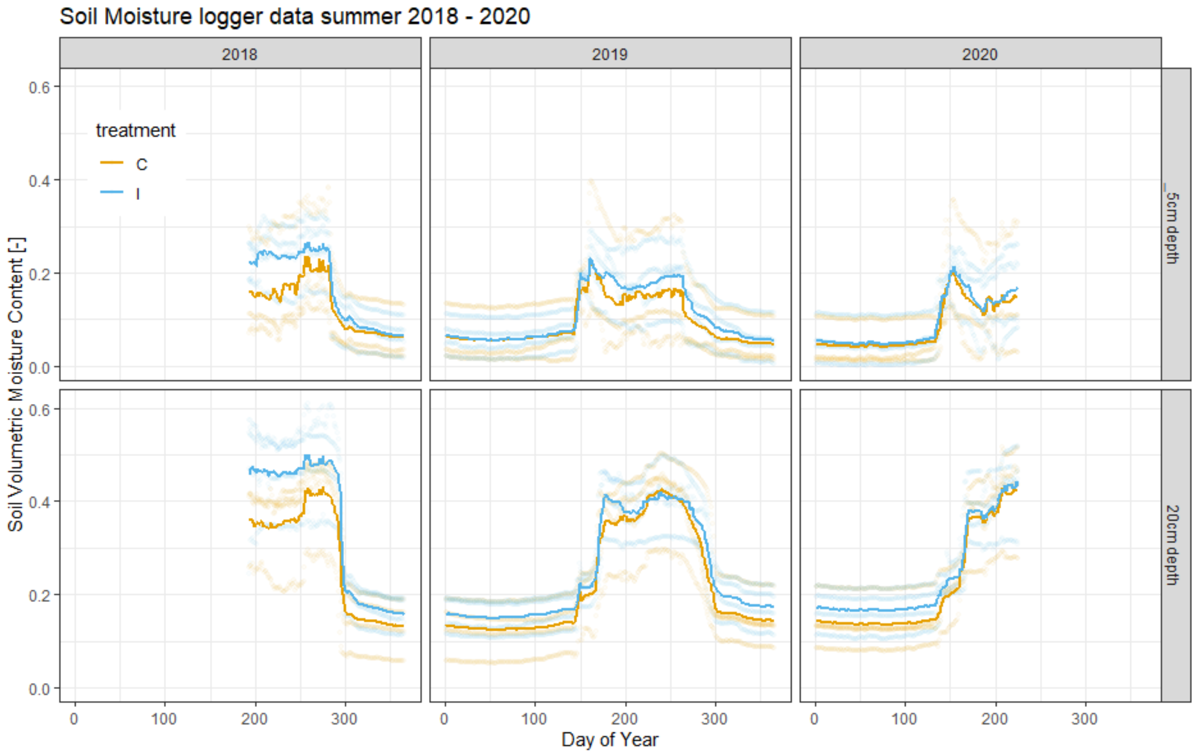

Supplementary Figure 4) *Field-measured daily volumetric soil moisture for irrigated (blue) and control (orange) plots at 5cm and 20cm depth from* S-SMC-005 *loggers. Transparent dots show individual logger measurements and opaque lines show averages per depth and treatment (n=4).*

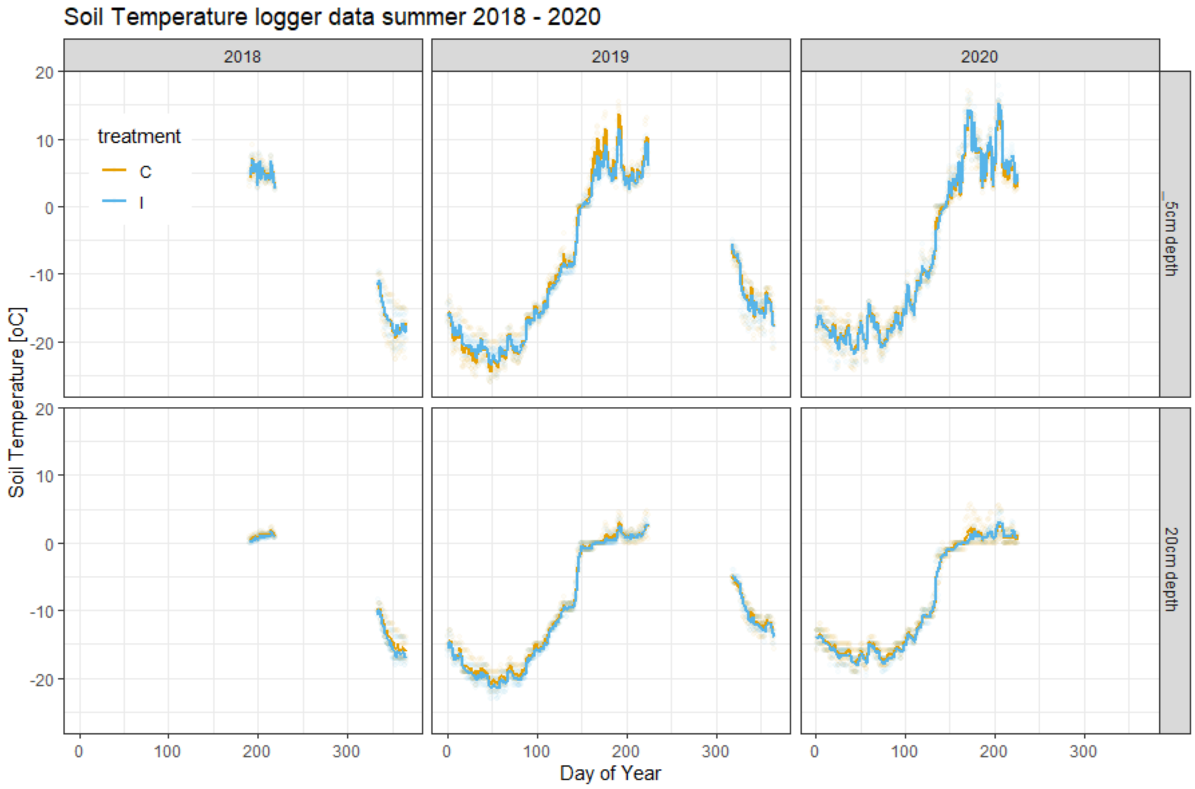

Supplementary Figure 5) *Field-measured daily soil temperatures for irrigated (blue) and control (orange) plots at 5cm and 20cm depth from iButton loggers. Transparent dots show individual logger measurements and opaque lines show averages per depth and treatment (n=4). Due to technical issues, field measured data show gaps in autumn.*

Supplementary Figure 4-5 show field-measured soil temperatures and soil volumetric moisture content at 5cm and 20cm depth. Temperature differences between irrigation and control are generally small. After irrigation, irrigated sites show warmer topsoil but colder subsoils in winter. The year after irrigation (2019), irrigated sites show colder topsoil in early summer. Higher soil moisture was observed in irrigated plots up until summer 2020. This difference was more pronounced in the subsoil (20cm depth).

Supplementary Results III - Experiment Replication using ATS

Model results are based on the input parameter combinations given in Supplementary Table 11 as well as the various forcing datasets containing daily atmospheric data.

Supplementary Table 11) Model parameters specified in the input files of ATS after configuration for site-specific conditions

Parameter	Moss	Organic	Miner al	Source	Calibration range
Bulk density (kg m^{-3})	75	88	1300	Field measured and[12]	-
Porosity (-)	0.93	0.94	0.51	Calculated from bulk density*	-
Permeability (m^2)	$1.7x10^{-11}$	$1.3x10^{-11}$	$6x10^{-13}$	[11]	-
van Genuchten alpha α (m^{-1})	$2.30x10^{-3}$	$1.42x10^{-3}$	$4.5x10^{-4}$	Calibrated	[9,11,24,25]
van Genuchten *m* (-)	0.22	0.275	0.17	Calibrated	[9,11,24,25]
Thermal conductivity κ (saturated) (W m^{-1} K^{-1})	0.6	0.5	2.55	Calibrated	[9,11,24-26]
Thermal conductivity κ (dry) (W m^{-1} K^{-1})	0.07	0.035	0.435	Calibrated	[9,11,24-26]
Surface roughness length (m)	0.6			Field measured vegetation height	-
Maximum ponded depth (m above soil surface)	0			Field measured water levels	
Bottom boundary temperature (K)	-263.65			[14]	-

*) Assuming average solid density of 2.65 g cm^{-3} for mineral and 1.4 g cm^{-3} for organic material.

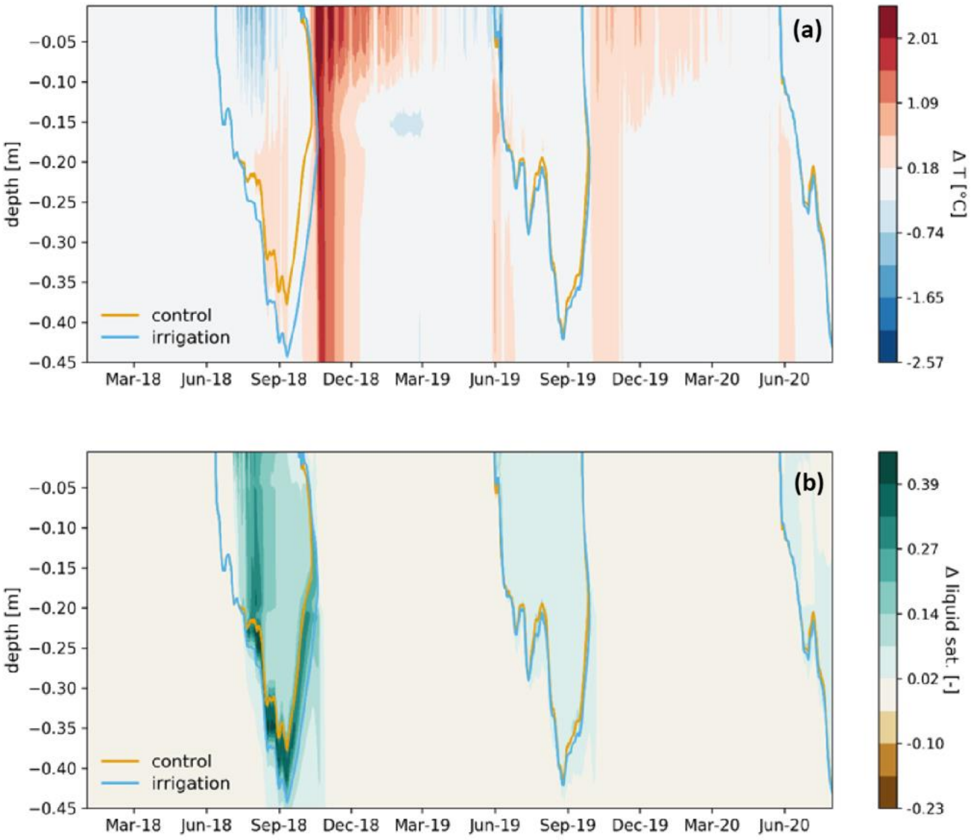

Supplementary Figure 6) a) *Daily temperature differences (irrigation-control) in the upper 0.45m of the subsurface for the experiment replication. Blue and orange contours represent the irrigation and control 0° isotherm respectively.* ***b)*** *Daily differences (irrigation-control) in soil liquid saturation in the upper 0.45m of soil for the experiment replication. Blue and orange contours represent the irrigation and control 0° isotherm respectively.*

Supplementary Figure 6 shows the depth of the 0°C isotherm and daily modelled temperature difference in the subsurface up to 0.45m depth. Supplementary Figure 7 shows daily modelled differences in soil liquid saturation. Irrigation was modelled to decrease topsoil temperatures but increase temperatures at depth, ultimately leading to a deeper active layer and delayed freeze-up under irrigation. In the subsequent winter (October 2018 to March 2019), topsoil temperatures remain more than 1 degree warmer in the irrigated plots compared to the control plots. At depth, the irrigated soil remains warmer until December 2018, but then cools down to being slightly colder than the control case. In the summer following irrigation, the irrigated soil shows a cooling effect in the beginning of summer, likely due to higher latent heat demands. The subsoil shows a mild warming effect of previous year's irrigation. The second winter after irrigation (winter 2019-2020) shows a similar, but dampened effect as in 2018. In the topsoil (and in the beginning at depth as well) a warming effect of up to 0.5°C can be seen in the irrigated case. Until the end of the year, the soil below 0.1m depth is mostly similar in both cases (Supplementary Figure 6a). The irrigation treatment was modelled to lead to substantial increases in soil volumetric water content. Small increases in moisture content persisted during the two summers after irrigation throughout the soil profile (Supplementary Figure 6b).

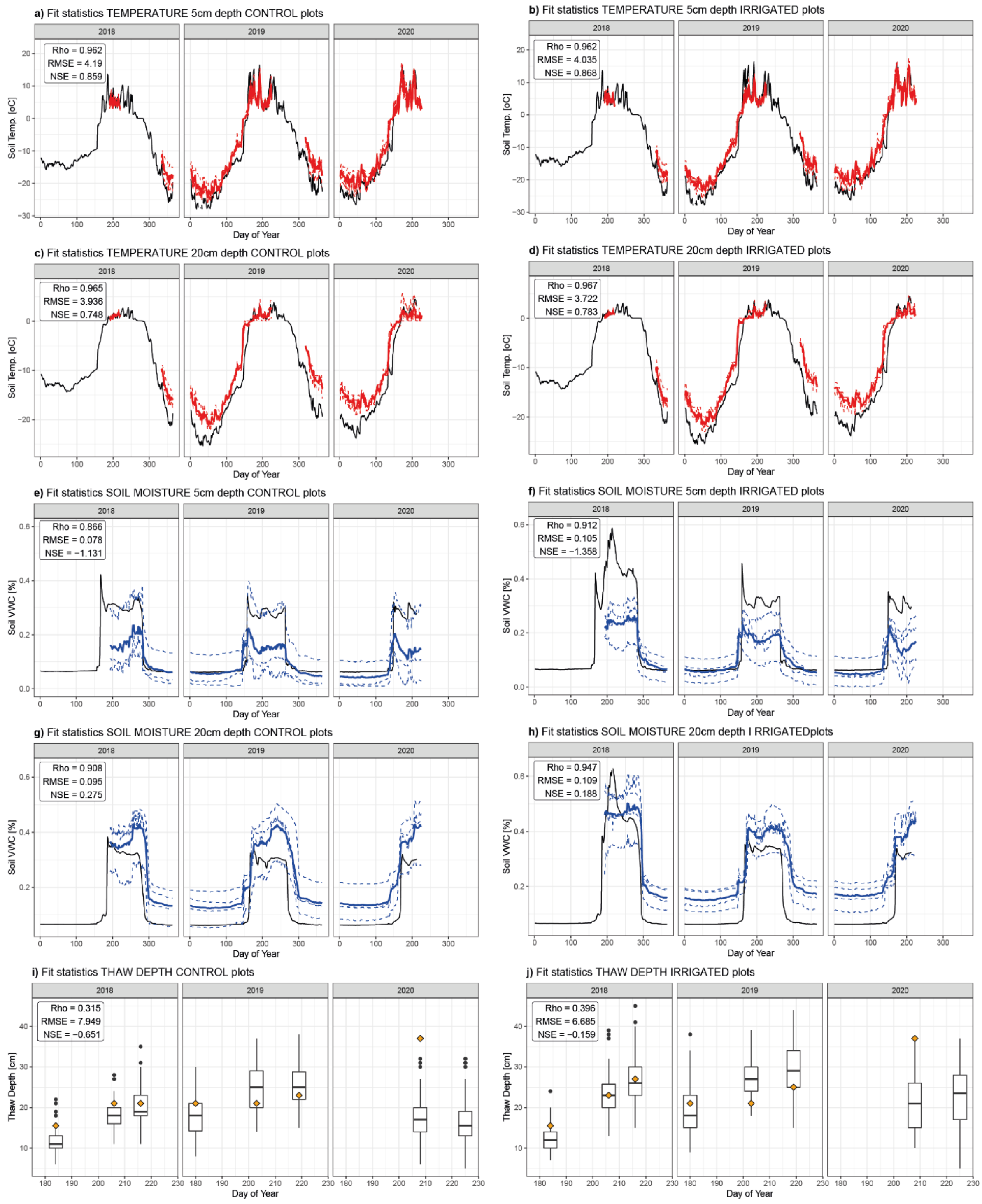

Supplementary Figure 7) *Fit statistics for field measured and modelled soil temperature, soil moisture and thaw depth in control plots and irrigated plots.* ***a)*** *Soil temperature at 5cm depth in control plots.* ***b)*** *Soil temperature at 5cm depth in irrigated plots.* ***c)*** *Soil temperature at 20cm depth in control plots.* ***d)*** *Soil temperature at 20cm depth in irrigated plots.* ***e)*** *Soil volumetric moisture content at 5cm depth in control plots.* ***f)*** *Soil volumetric moisture content at 5cm depth in irrigated plots.* ***g)*** *Soil volumetric moisture content at 20cm depth in control plots.* ***h)*** *Soil volumetric moisture content at 20cm depth in irrigated plots.* ***i)*** *Thaw depth in control plots.* ***j)*** *Thaw depth in irrigated plots. In* ***a-h****, coloured dashed lines represent individual sensor series and coloured solid lines represent averages per treatment and*

*depth. Black lines represent modelled values. In **(i-j)**, boxplots represent treatment averages for all field measurements (n=90 per box) per measurement moment and points represent model predicted thaw depth on the same day. In boxplots, centre lines represent the median, box limits represent upper and lower quartiles, whiskers represent 1.5 times the interquartile range and points represent outliers.*

In Supplementary Figure 8a, modelled temperatures at 5 and 20cm depth are compared to differences in averaged temperatures measured in irrigated plots and control plots. An irrigation effect is visible in both modelled and field temperatures, although differences in magnitude exist. Due to logger-failure, summer temperatures were not recorded in August-October, but were likely warmer in the irrigated site vs. the control site given the deeper active layer. After freeze-up, the effects described above are visible in both records; warming in the topsoil until spring 2019 and slightly colder temperatures at depth in winter until early summer 2019. Further, in summer 2019, the topsoil in the measurements also remains colder, up to 2.5°C, while the modelled effect only amounts to about 1°C temperature difference. At depth, summer temperature differences in 2019 show a good agreement between both records moving around 0°C differences.

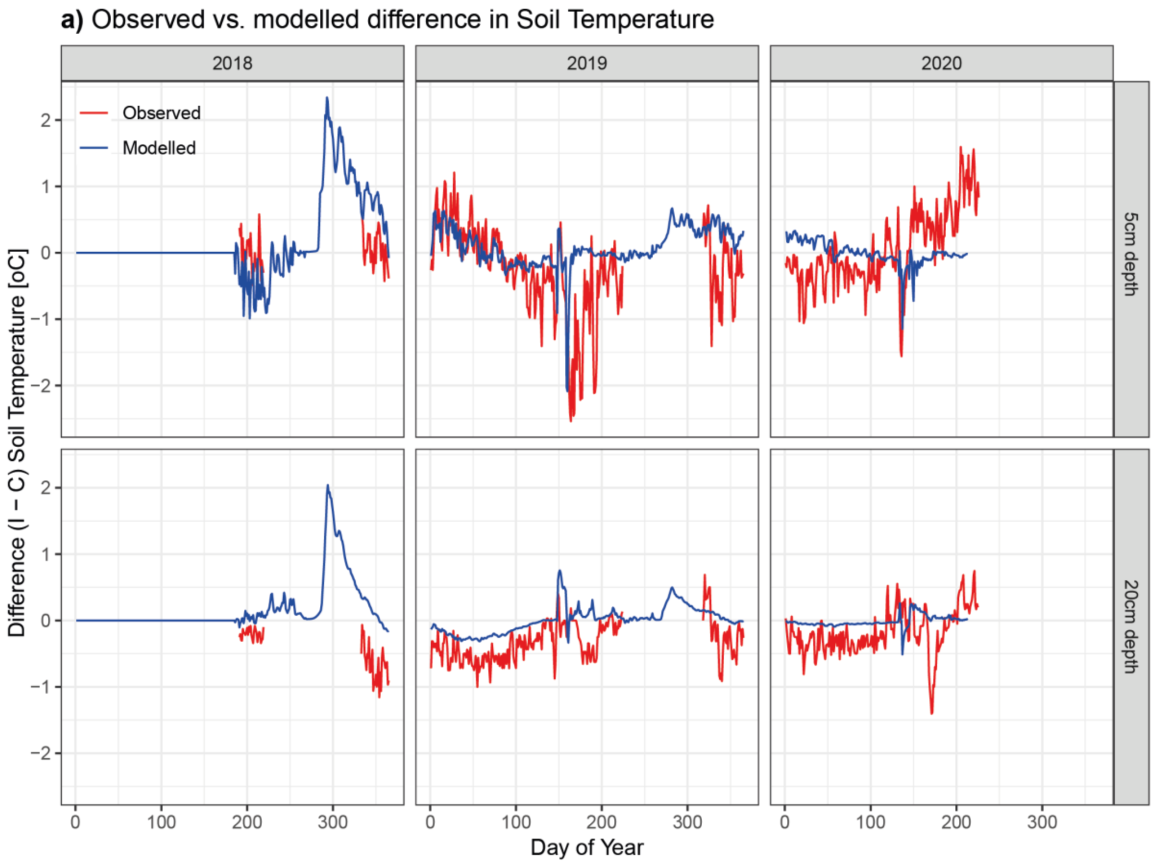

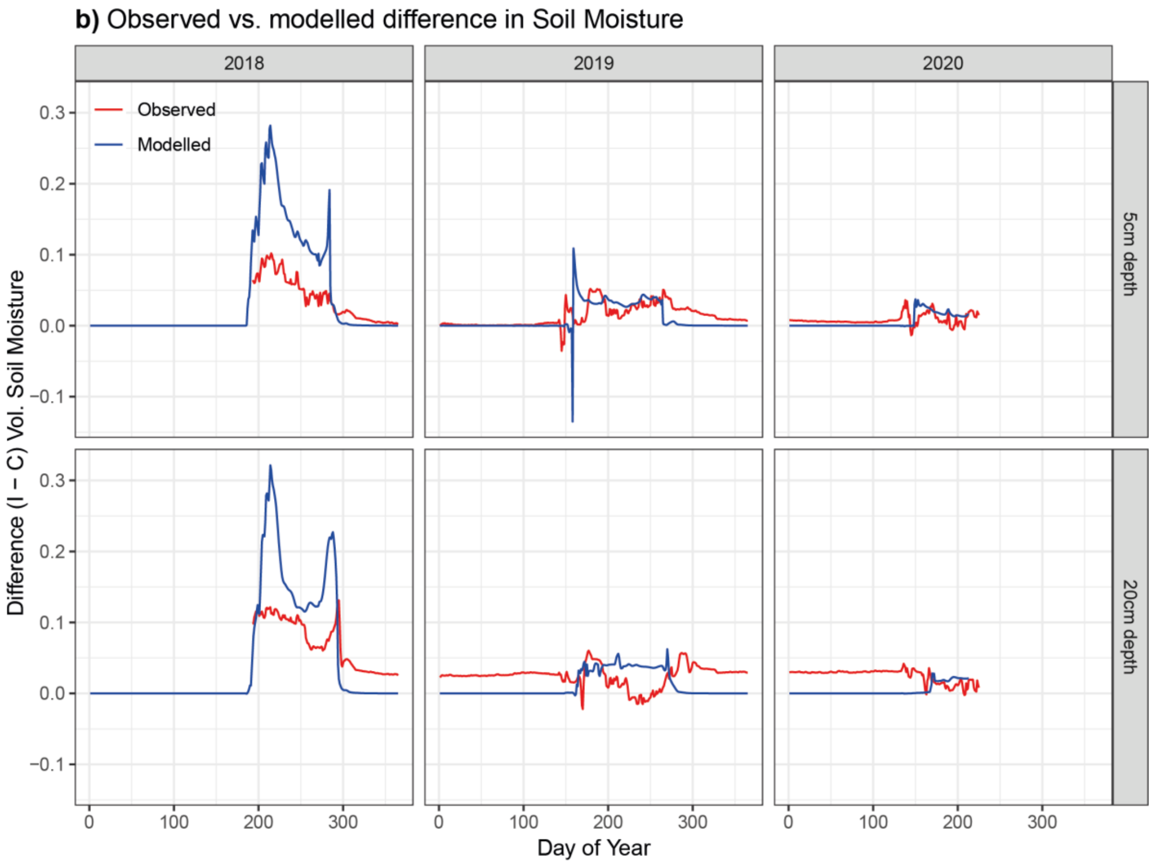

Supplementary Figure 8) a) *Measured (red) and modeled (blue) temperature differences between irrigation and control at 5 and 20cm depth from 2018 to 2020. Modeled values represent temperatures*

at 5cm and 20cm depth for a single column soil model. Measured differences represent the difference between averaged temperature in irrigated (n = 4) - control (n = 4) plots at approximately 5cm and 20cm depth, measured with iButtons. (see "Field Measurements"). Due to technical issues, iButton records show gaps in autumn. ***b)*** *Soil Moisture differences between measured (red) and modeled (blue) temperatures in 5 and 20cm depth from 2018 to 2020. Modeled values represent temperatures at 5cm and 20cm depth for a single column soil model. Measured differences represent the difference between averaged temperature in irrigated (n = 4) - control (n=4) plots at approximately 5cm and 20cm depth, measured with EC5 sensors. (see "Field Measurements").*

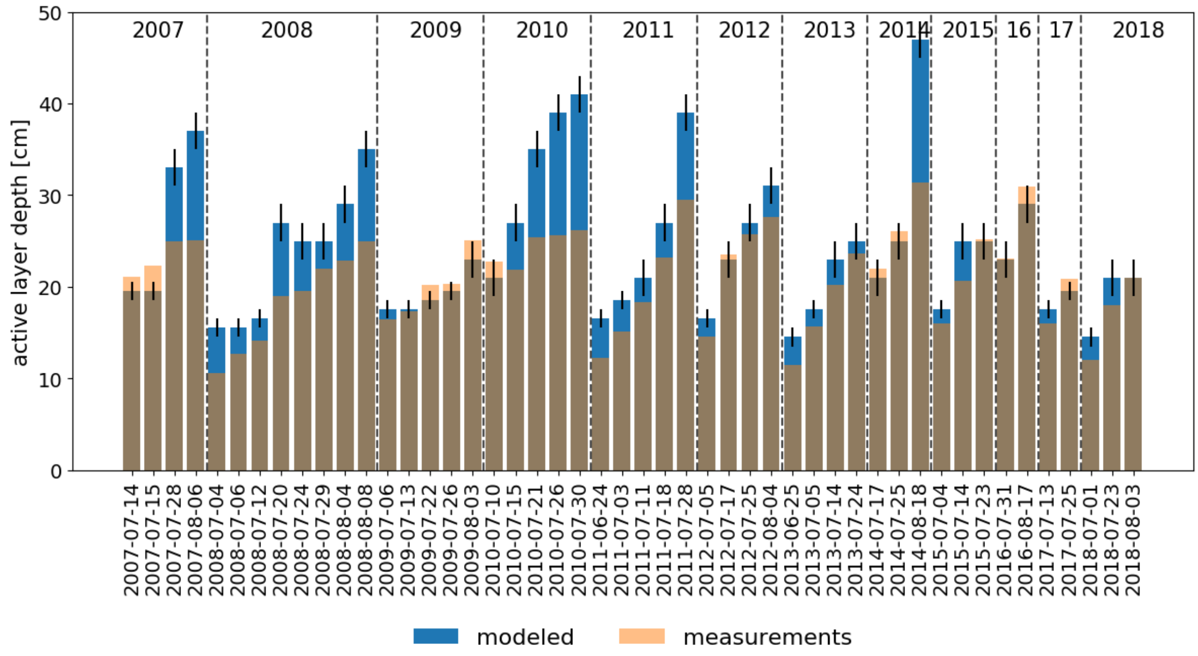

Supplementary Figure 9) *Model predicted historical thaw depths using calibrated parameters compared to historical measurements in undisturbed shrub tundra at approximately 500m distance from the irrigated sites[16-18] represented by the model. Error bars for modelled values represent potential model error resulting from cell discretization (cells are 1 – 2 cm thick depending on depth, see Supplementary Methods III). Modelled values show dynamics similar to field measurements (Spearman's rho = 0.87, p value < 1E^{-14}, NSE = 0.44), although substantial offsets occur in some years (RMSE = 5.75cm), where modelled values exceed field measured values. Although the sites of the historical measurement series and the irrigated sites upon which the model calibration is based showed similar conditions and vegetation, differences among these sites may have contributed to observed differences between measured and modelled values.*

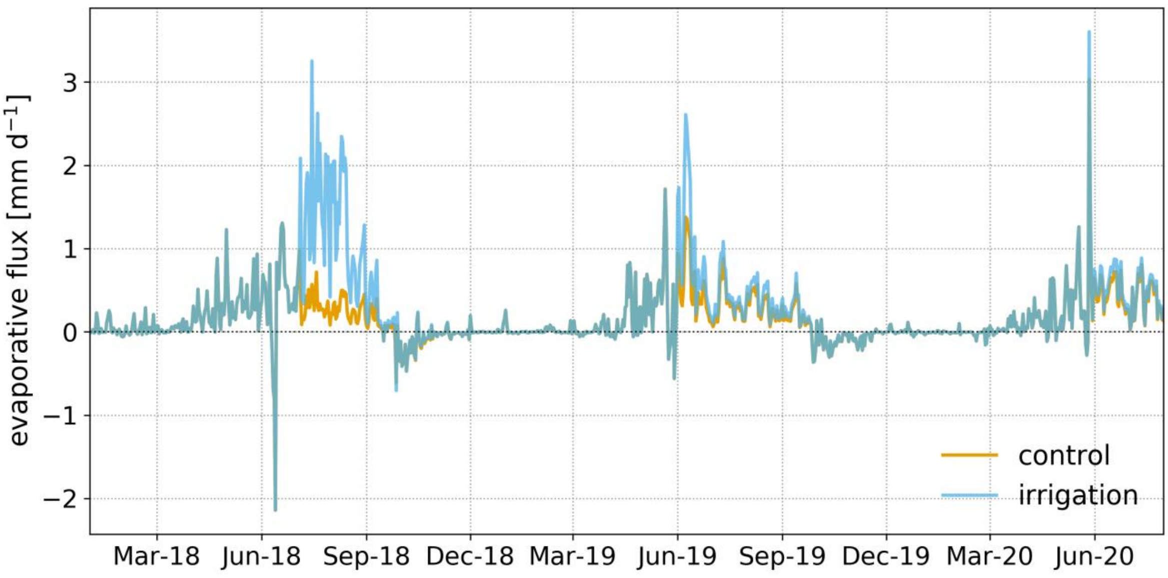

Supplementary Figure 10) *Simulated surface evaporative flux in mm d^{-1} for the control (orange) and irrigated (blue) case for the experiment replication.*

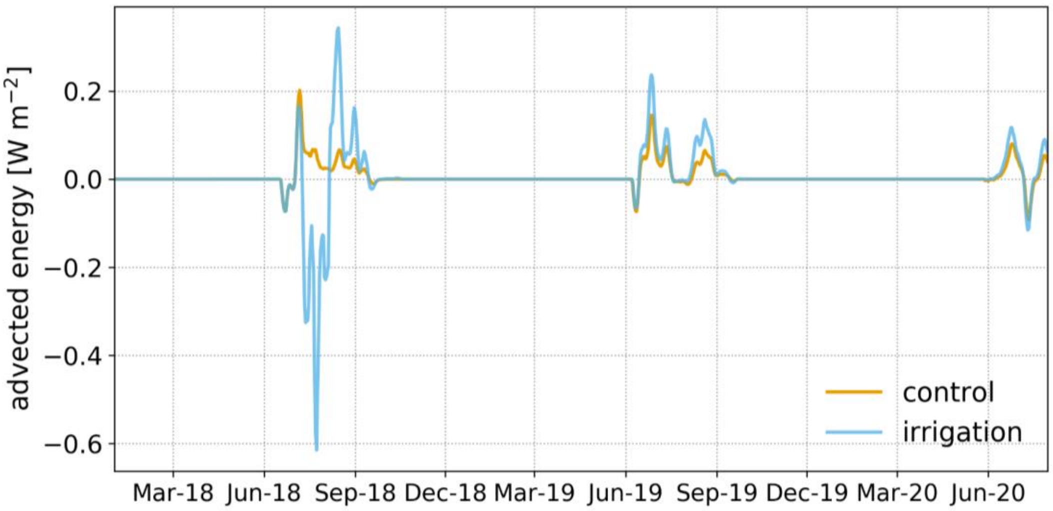

Supplementary Figure 11) *Simulated daily vertical advected energy flux in 0.1m depth (9-day moving average) for the control (orange) and irrigated (blue) case for the experiment replication. Positive fluxes indicate heat flux from topsoil to the atmosphere, negative fluxes indicate heat flux from atmosphere to topsoil.*

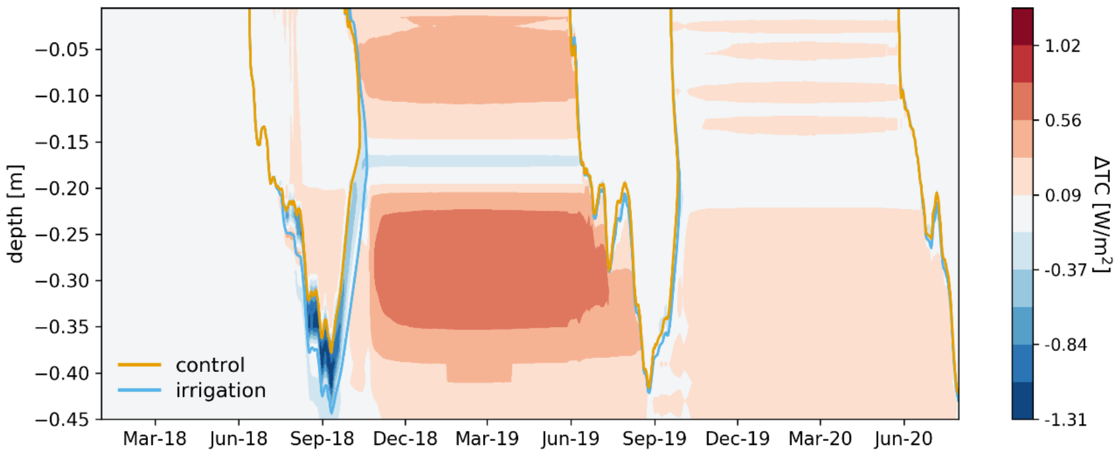

Supplementary Figure 12) *Simulated daily difference (irrigation - control) in bulk thermal conductivity in the top 0.45m of soil for the experiment replication. Blue and orange contours represent the irrigation and control 0° isotherm respectively.*

Supplementary Figure 10-12 show modelled differences in evaporative flux, advected heat flux and thermal conductivity between control and irrigated plots. A substantial amount of the added water during the experiment starts evaporating right after irrigation (Supplementary Figure 10). While evaporative fluxes in the control case rarely exceed 1mm d^{-1}, evaporation in the irrigated cases reaches up to 3mm d^{-1}. In total, evaporation in 2018 amounts to 53.3mm in the control, and 122.9mm in the irrigated case, corresponding to a 2.3-fold increase. In the second year, evaporation is still higher in the irrigated site, indicating that additional water remained frozen in pore space during winter. With 71.2mm, evaporation in the irrigated case is only 25% higher than in the control case (56.7mm). On the other hand, energy advected by percolating water can be found after irrigation (Supplementary Figure 11). This indicates that mass, and with it energy, is being transported into deeper soil layers due to irrigation. As a result, thermal conductivity of the soil is increased, especially in subsequent winters in mineral soil layers (Supplementary Figure 12).

Supplementary Results IV – Sensitivity Analysis

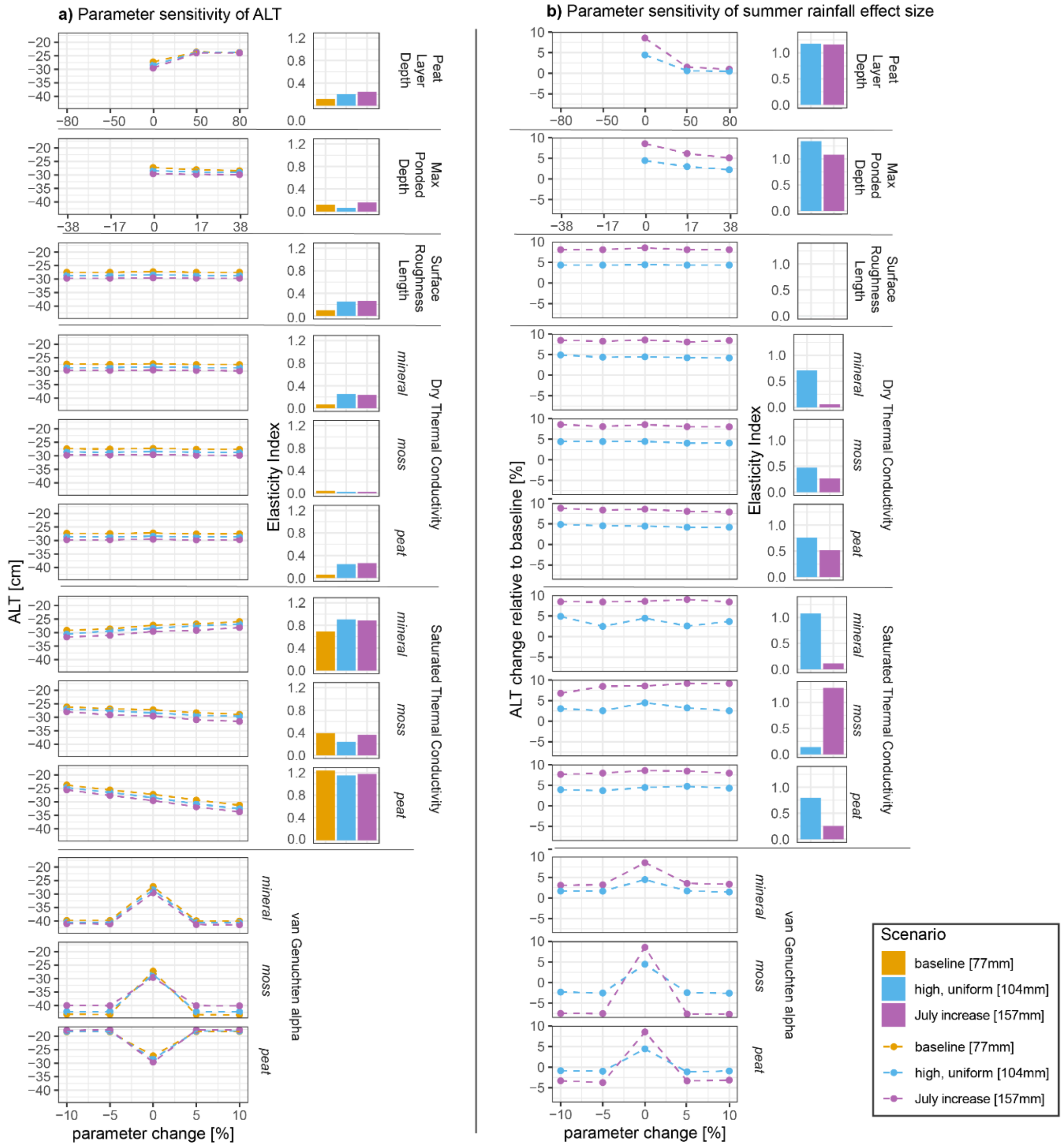

***Supplementary Figure 13) a**) Sensitivity of ALT to soil stratigraphical, microtopographical, thermal and hydrological parameters. Missing values in the line plots indicate runs with altered parameter values that did not converge. The Elasticity Index is calculated as the percentual change in ALT over the parameter space relative to the percentual change in the parameter. **b**) Sensitivity of summer rainfall effect on ALT (calculated % increase in ALT compared to baseline scenario) to soil stratigraphical, microtopographical, thermal and hydrological parameters. The Elasticity Index is calculated as the percentual change in ALT change relative to the baseline scenario over the parameter space relative to the percentual change in the parameter. In both **a** & **b**, van Genuchten soil water retention parameter alpha shows a non-linear effect on ALT dynamics, which is why no elasticity index was computed.*

Model sensitivity (Supplementary Figure 13) indicates that the saturated thermal conductivity of the peat layers has a high proportional influence on ALT, arguably because this is the thickest unfrozen

layer throughout the summer season. Although proportional changes in ALT under variable peat layer thickness and maximum ponded depth were low, these stratigraphical and microtopographical parameters may vary substantially on small spatial scales[19] and thereby still exert substantial influence on ALT. ALT shows a strongly non-linear response to van Genuchten water retention parameters. Changing hydraulic properties in the peat layer for instance can determine whether thaw penetrates to the mineral soil layer (depth of 20cm or more) or remains confined to the peat layer, showing as an abrupt change in ALT and in the effect size of rainfall (Supplementary Figure 13). The effect size of rainfall also decreased under increasing ponded depth (suggesting lower topographical positions where water can accumulate above the surface), although the effect of microtopography is difficult to incorporate in a 1D-column model as effects of lateral flow are neglected. These outcomes suggest that the size of summer rainfall effects on ALT depends on local stratigraphical, hydrological and microtopographical differences, but likely most importantly on the thickness and thermal-hydrological properties of organic topsoils.

Non-linear responses to van Genuchten parameters may also be related to the interrelatedness of various soil physical properties such as textural composition and soil hydrological and thermal parameters. In this sense, changing only one parameter at a time may not be very representative of real-world physical differences among soils. Supplementary Figure 14 gives an overview of scenario outcomes for several standard soil textural classes with corresponding hydrological and thermal parameters.

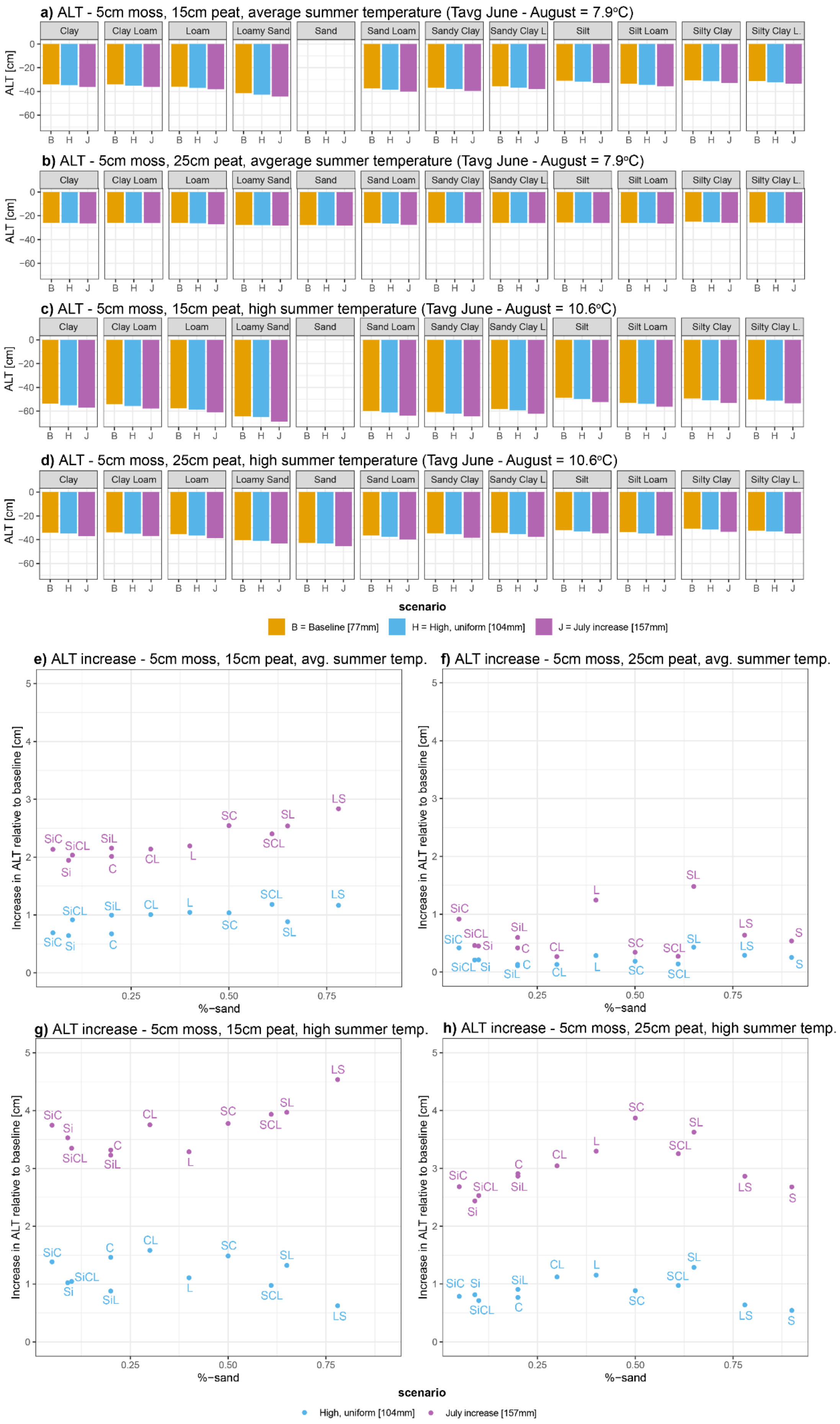
a) ALT - 5cm moss, 15cm peat, average summer temperature (Tavg June - August = 7.9°C)
b) ALT - 5cm moss, 25cm peat, avgerage summer temperature (Tavg June - August = 7.9°C)
c) ALT - 5cm moss, 15cm peat, high summer temperature (Tavg June - August = 10.6°C)
d) ALT - 5cm moss, 25cm peat, high summer temperature (Tavg June - August = 10.6°C)
Clay
Clay Loam
Loam
Loamy Sand
Sand
Sand Loam
Sandy Clay
Sandy Clay L
Silt
Silt Loam
Silty Clay
Silty Clay L.
ALT [cm]
scenario
B = Baseline [77mm]
H = High, uniform [104mm]
J = July increase [157mm]
e) ALT increase - 5cm moss, 15cm peat, avg. summer temp.
f) ALT increase - 5cm moss, 25cm peat, avg. summer temp.
g) ALT increase - 5cm moss, 15cm peat, high summer temp.
h) ALT increase - 5cm moss, 25cm peat, high summer temp.
Increase in ALT relative to baseline [cm]
%−sand
scenario
High, uniform [104mm]
July increase [157mm]

Supplementary Figure 14) *Effects of rainfall on ALT across soil textural types, peat layer thickness and summer temperature.* ***a)*** *ALT per against mineral soil texture for the 12 USDA soil texture types under moss and peat layer thickness representative for the field site and average field site summer temperature (Tavg JJA = 7.9 °C).* ***b)*** *ALT per against mineral soil texture for the 12 USDA soil texture with increased peat layer thickness (25cm) and average field site summer temperature.* ***c)*** *ALT per against mineral soil texture for the 12 USDA soil texture types under moss and peat layer thickness representative for the field site and very high summer temperature (Tavg JJA = 10.6 °C) relative to field site average.* ***d)*** *ALT per against mineral soil texture for the 12 USDA soil texture types with increased peat layer thickness and very high summer temperature.* ***e)*** *ALT increase relative to baseline scenario for each soil texture type under moss and peat layer thickness representative for the field site and average field site summer temperature and average field site summer temperature.* ***f)*** *ALT increase relative to baseline scenario for each soil texture type under moss and peat layer thickness representative for the field site and very high summer temperature.* ***g)*** *ALT increase relative to baseline scenario for each soil texture type under increased peat layer thickness and average field site summer temperature.* ***h)*** *ALT increase relative to baseline scenario for each soil texture type under increased peat layer thickness and very high summer temperature. In* ***e-h*** *S = Sand, LS = Loamy Sand, SL = Sand Loam, SCL = Sandy Clay Loam, SC = Sandy Clay, L = Loam, CL = Clay Loam, C = Clay, SiL = Silt Loam, SiCL = Silty Clay Loam, Si = Silt, SiC = Silty Clay. In* ***a & c****, the runs for sand did not convergence and hence no results are available.*

Under the field site conditions, larger ALTs and larger ALT increases under increased rainfall were modelled if soil physical, hydrological and thermal parameters (Supplementary Table 3) were set to represent coarser texture mineral soil layers (Supplementary Figure 14). Under increased peat layer thickness, these contrasts were attenuated to a large degree, likely since thaw did not penetrate into the mineral soil layers deeply and was mostly determined by the properties of the moss and peat layers. If increased peat layer thickness was accompanied by warmer summer temperatures, larger differences in ALT and ALT increase under increased rainfall were modelled among soil textural classes. In this case especially moderately coarse textured soils (Loam, Sand Loam, Sandy Clay and Sandy Clay Loam) showed large increases in ALT under increased rainfall. Under enhanced summer temperatures, ALT was especially increased under rainfall extremes during mid-summer, whereas more moderate and uniformly distributed rainfall increases showed small effects on ALT across mineral soil types. This suggests that extreme precipitation events especially increase ALT during warm summers in soils with coarse sediments underlying shallow organic layers, whereas little difference may be evident across soil textural types under thick peat layers and uniform and smaller increases in precipitation.

Supplementary Results IV - Scenario Results

.

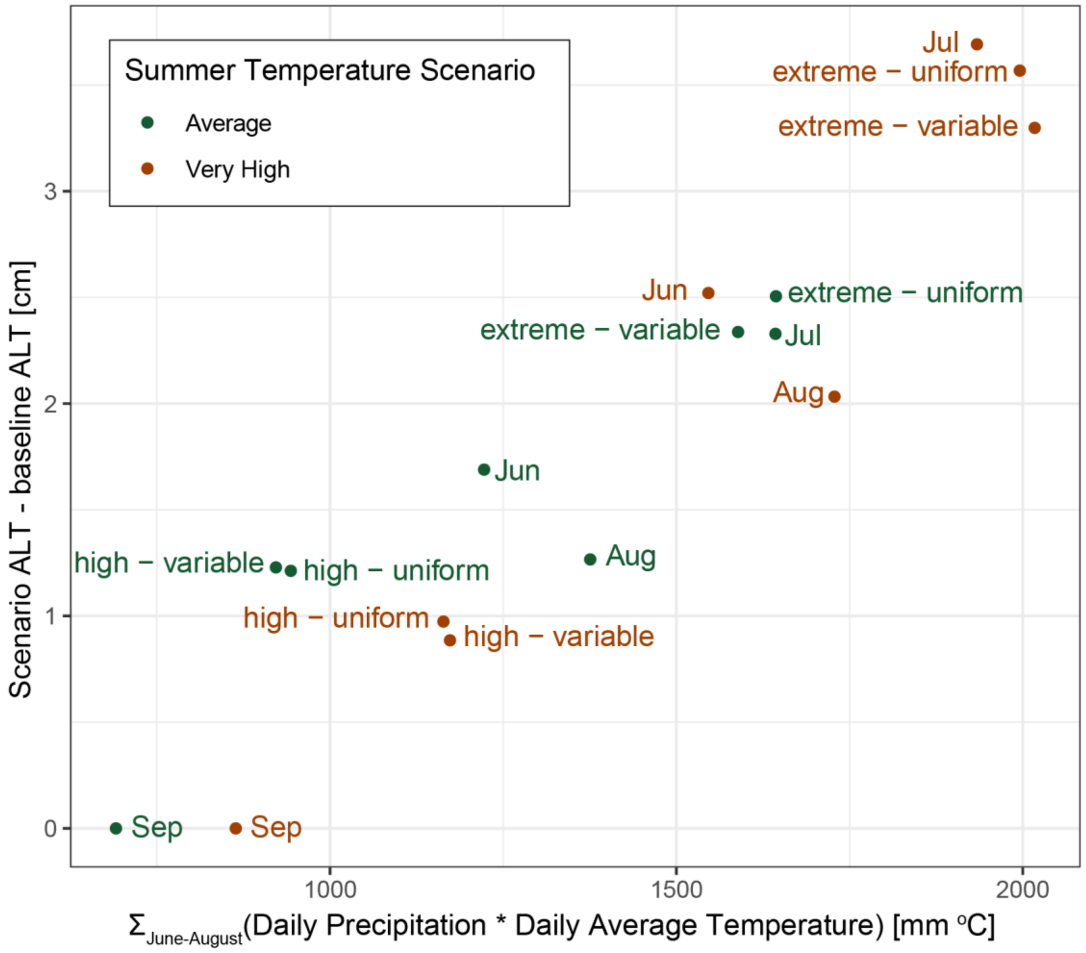

Supplementary Figure 15) *Relation between observed difference between scenario ALT (first year only) and sum of the product of scenario daily precipitation and daily temperature between June 1st and August 31st. Labels indicate precipitation scenarios, colours indicate temperature scenario (green = summer with average temperature, orange = summer with very high temperature).*

Supplementary Figure 15 indicates that the modelled difference in ALT between baseline and increased precipitation scenarios shows strong linearity with the product of daily precipitation and daily average temperatures. This indicates that the effect of summer rainfall on ALT likely depends strongly on co-occurrence of precipitation and temperature peaks.

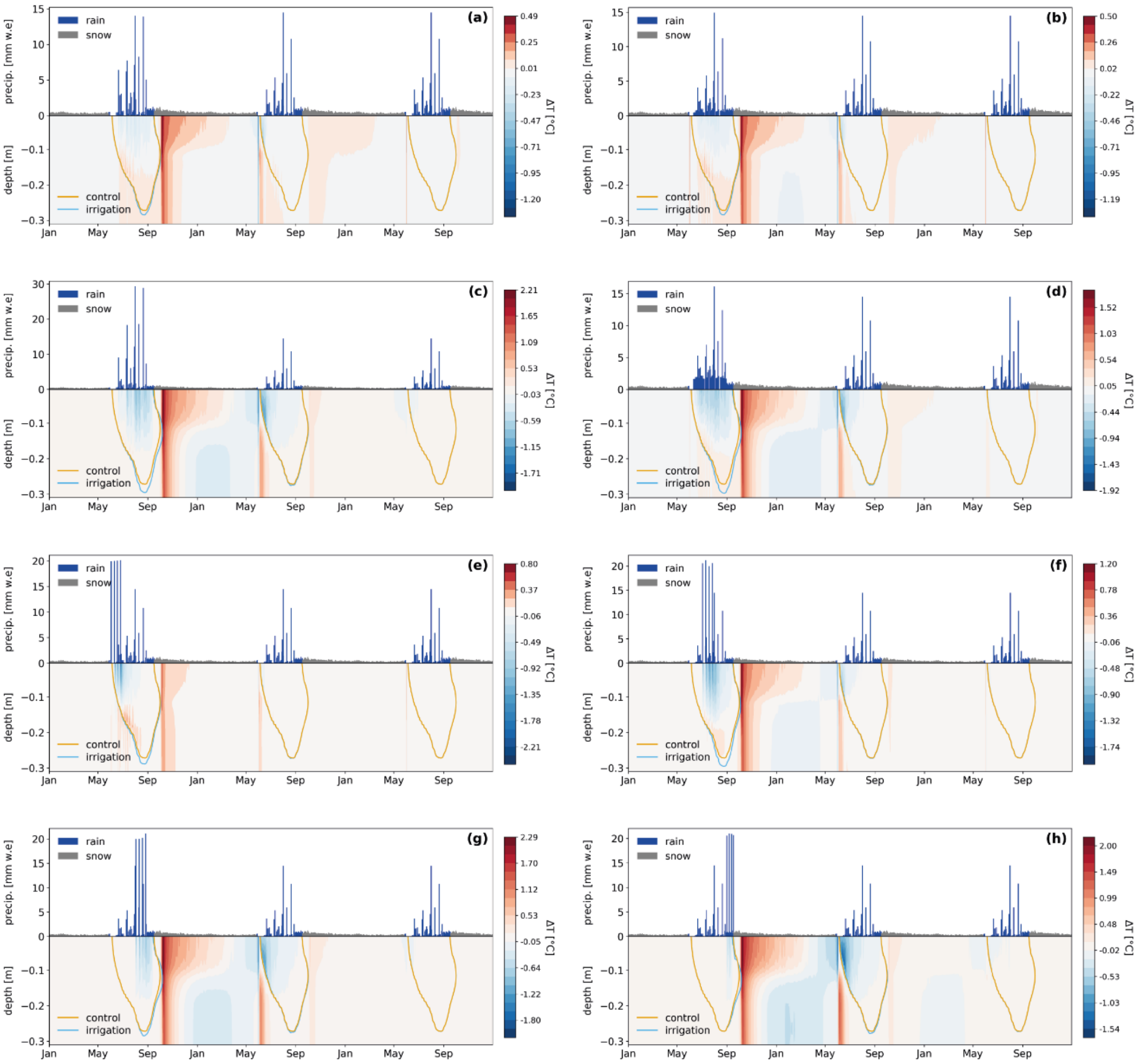

Supplementary Figure 16) *Simulated daily soil temperature difference with baseline (scenario - baseline temperature) for scenarios and two following years with baseline precipitation in a summer with average temperatures (Tavg JJA = 7.9 °C).* ***a)*** *high precipitation scenario with variable increase over June-August.* ***b)*** *high precipitation scenario with uniform increase over June-August.* ***c)*** *extreme precipitation scenario with variable increase over June-August.* ***d)*** *extreme precipitation scenario with uniform increase over June-August.* ***e)*** *June extreme precipitation scenario.* ***f)*** *July extreme precipitation scenario.* ***g)*** *August extreme precipitation scenario.* ***h)*** *September extreme precipitation scenario.*

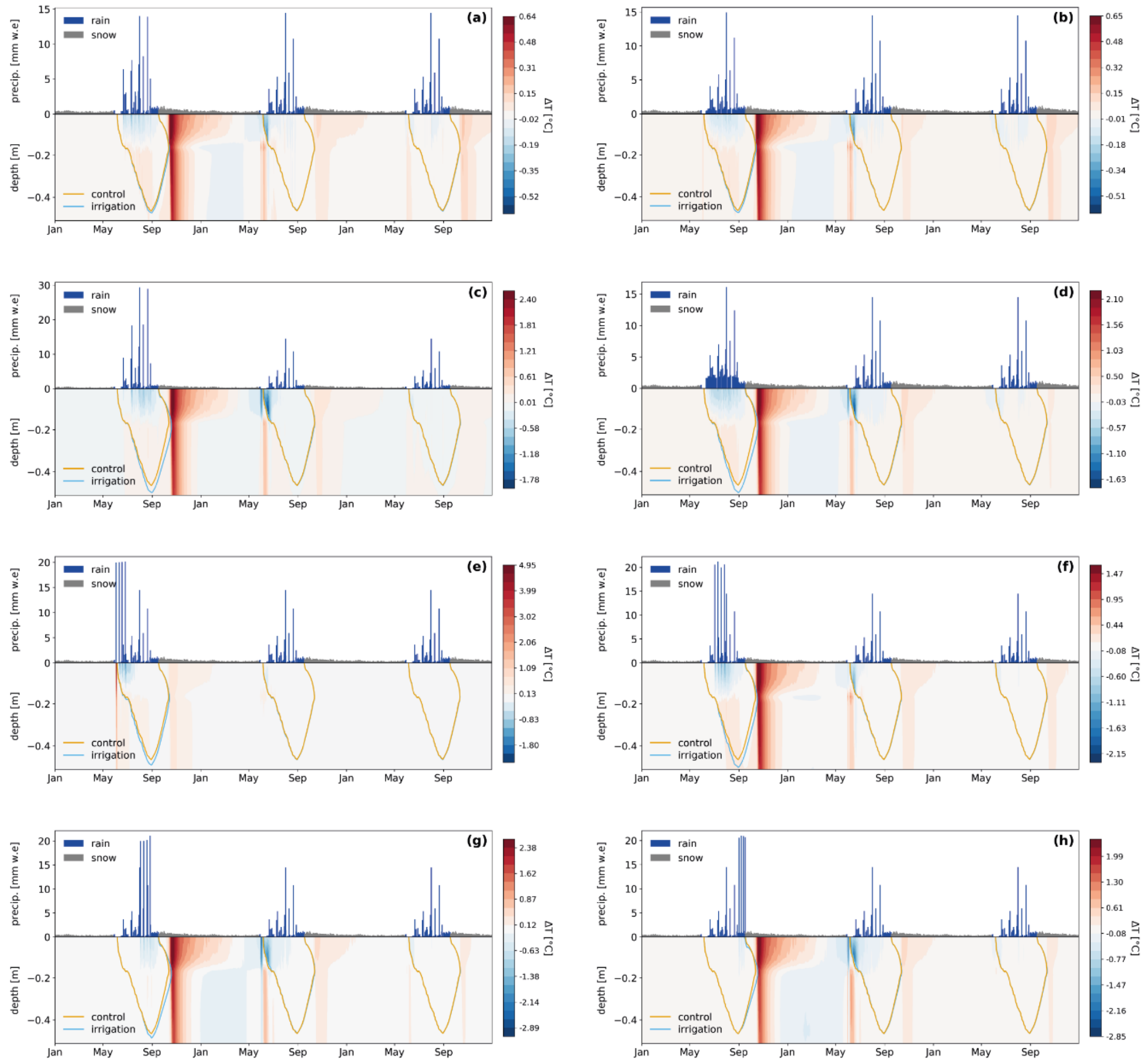

Supplementary Figure 17) *Simulated daily soil temperature difference with baseline (scenario - baseline temperature) for scenarios and two following years with baseline precipitation in a summer with very warm temperatures (Tavg JJA = 10.6 °C).* ***a)*** *high precipitation scenario with variable increase over June-August.* ***b)*** *high precipitation scenario with uniform increase over June-August.* ***c)*** *extreme precipitation scenario variable increase over June-August.* ***d)*** *extreme precipitation scenario with uniform increase over June-August.* ***e)*** *June extreme precipitation scenario.* ***f)*** *July extreme precipitation scenario.* ***g)*** *August extreme precipitation scenario.* ***h)*** *September extreme precipitation scenario.*

Supplementary Figure 16 – 17 show scenario results for site averaged conditions (peat thickness = 15cm, maximum ponded depth 0cm above surface).

Supplementary Results V – Miscellaneous

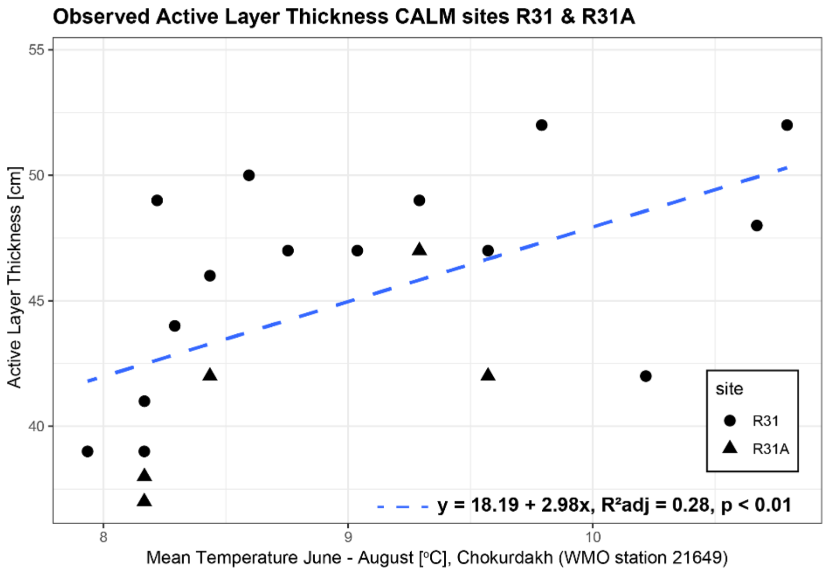

Supplementary Figure 18) *Annual observed Active Layer Thickness in monitoring sites of the Circumarctic Active Layer Monitoring (CALM) programme. Sites R31 (Yedoma ridge, 70.55N, 147.43E) and site R31A (alas, 70.57N, 147.42E) are situated close to Chokurdakh (70.38N, 147.54E) and are the monitoring sites closest to our study site. Data from Abramov et al. (2019)*[27].

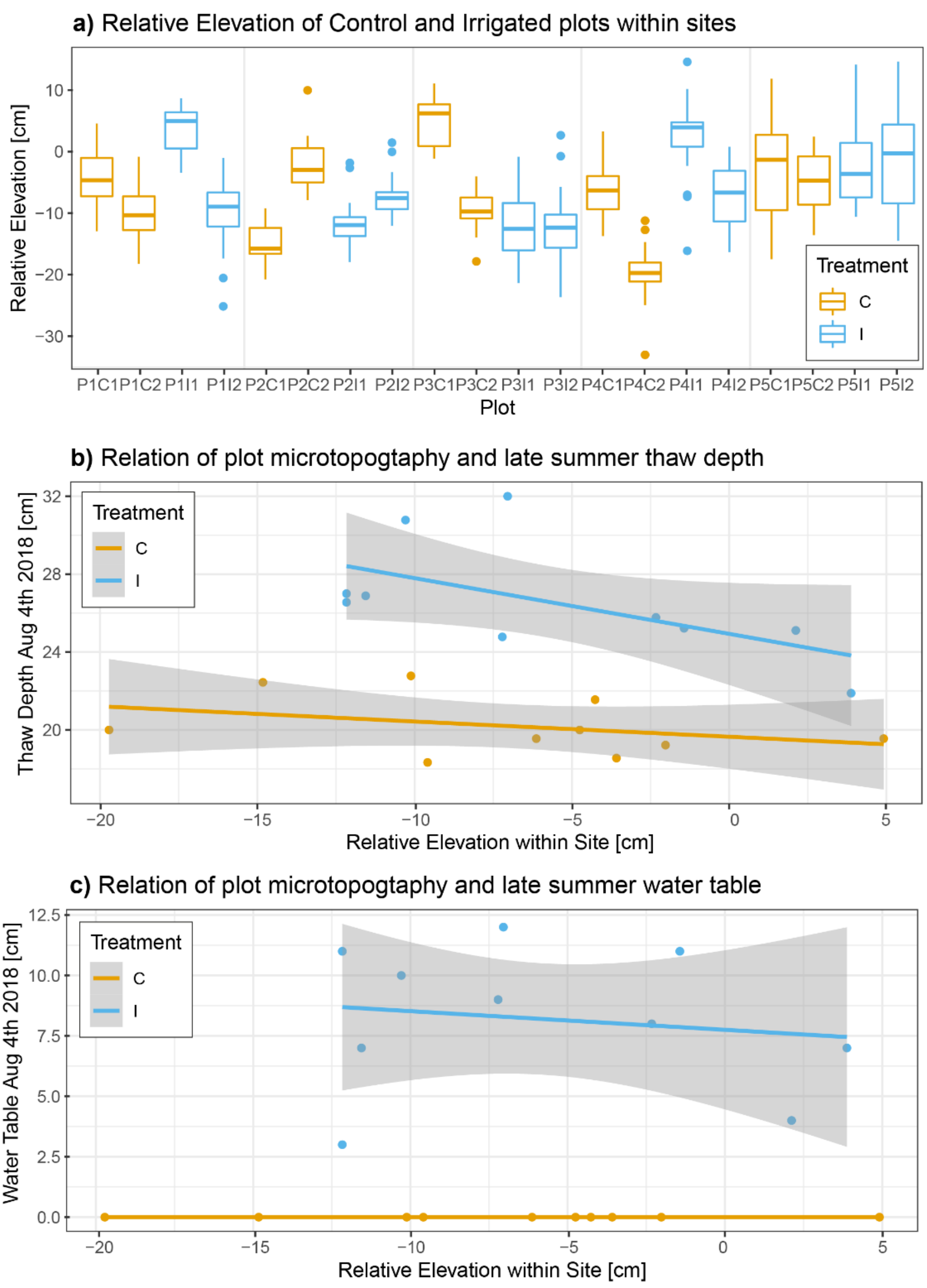

Supplementary Figure 19) a*) Relative Elevation per plot, standardized to mean outside plot elevation per site (P1-P5). Centre lines represent the median, box limits represent upper and lower quartiles, whiskers represent 1.5 times the interquartile range and points represent outliers. No structural difference between control and irrigated sites was evident (F=0.168, ddf=18, p=0.687).* ***b)*** *Relation between average Relative Elevation per plot and average Thaw Depth on August 4th, 2018. No significant overall relation was found between Relative Surface Elevation and Thaw Depth. A weak negative relation between a plot's relative surface elevation within a site and average thaw depth per plot seems discernible in irrigated sites, although it was not significant (Supplementary Table 12).* ***c)*** *Relation between average Relative Elevation per plot and Water Table on top of the permafrost in the plot centre on August 4th 2018. No significant overall relation was found between a plot's relative surface elevation within a site and height of the water table above the permafrost (Supplementary*

*Table 13). In **b** and **c**, shaded areas represent a 95% confidence interval around the estimated linear fit.*

Supplementary Table 12) Coefficient estimates for linear models of averaged August thaw depth per plot as a function of treatment and average plot Relative Elevation.

Model Specification: *Thaw Depth August 2018 ~ Relative Elevation * Treatment*

Coefficient	*Estimate*	*Standard Error*	*t-value*	*p-value*
Intercept	19.652	0.964	20.387	<0.001***
Relative Elevation	-0.078	0.100	-0.778	0.447
Treatment	5.283	1.347	3.921	0.001**
Relative Elevation : Treatment	-0.208	0.153	-1.359	0.193

Full model adjusted R-squared: 0.7282, F-statistic: 17.97 on 3 and 16 df, p-value: 2.246e-05.

Supplementary Table 13) Coefficient estimates for linear models of averaged August Water Table in plot centres as a function of treatment and average plot Relative Elevation.

Model Specification: *Water Table August 2018 ~ Relative Elevation * Treatment*

Coefficient	*Estimate*	*Standard Error*	*t-value*	*p-value*
Intercept	$-3.093e^{-16}$	1.032	0.000	1.000
Relative Elevation	$-4.822e^{-17}$	0.108	0.000	1.000
Treatment	7.751	1.443	5.372	<0.001***
Relative Elevation : Treatment	$-7.711e^{-2}$	0.164	-0.471	0.644

Full model adjusted R-squared: 0.7736, F-statistic: 22.64 on 3 and 16 df, p-value: 5.316e-06.

References

1 Neumann, R. B. *et al.* Warming effects of spring rainfall increase methane emissions from thawing permafrost. *Geophysical Research Letters* **46**, 1393-1401 https://doi.org/10.1029/2018GL081274 (2019)

2 Byers, H. R., Moses, H. & Harney, P. J. Measurement of rain temperature. *Journal of Atmospheric Sciences* **6**, 51-55, https://doi.org/10.1175/1520-0469(1949)006<0051:MORT>2.0.CO;2 (1949)

3 All-Russia Research Institute of Hydrometeorological Information - World Data Centre (RIHMI-WDC). http://aisori-m.meteo.ru/waisori/ (2020).

4 Bates, D., Mächler, M., Bolker, B., & Walker, S. Fitting Linear Mixed-Effects Models Using lme4. Journal of Statistical Software, **67**, 1–48. https://doi.org/10.18637/jss.v067.i01 (2015)

5 R: A language and environment for statistical computing (R Foundation for Statistical Computing, Vienna, Austria, 2019).

6 Halekoh, U. & Højsgaard, S. A kenward-roger approximation and parametric bootstrap methods for tests in linear mixed models–the R package pbkrtest. *Journal of Statistical Software* **59**, 1-30. https://doi.org/10.18637/jss.v059.i09 (2014)

7 Brooks, M. E. *et al.* glmmTMB balances speed and flexibility among packages for zero-inflated generalized linear mixed modeling. *The R journal* **9**, 378-400. https://doi.org/10.3929/ethz-b-000240890 (2017)

8 Hartig, F. DHARMa: Residual Diagnostics for Hierarchical (Multi-Level / Mixed) Regression Models. R package version 0.1.5. http://florianhartig.github.io/DHARMa/ (2017)

9 Atchley, A. L., Coon, E. T., Painter, S. L., Harp, D. R. & Wilson, C. J. Influences and interactions of inundation, peat, and snow on active layer thickness. *Geophysical Research Letters* **43**, 5116-5123. https://doi.org/10.1002/2016GL068550 (2016)

10 Painter, S. L. *et al.* Integrated surface/subsurface permafrost thermal hydrology: Model formulation and proof-of-concept simulations. *Water Resources Research* **52**, 6062-6077. https://doi.org/10.1002/2015WR018427 (2016)

11 Jan, A., Coon, E. T. & Painter, S. L. Evaluating integrated surface/subsurface permafrost thermal hydrology models in ATS (v0. 88) against observations from a polygonal tundra site. *Geoscientific Model Development* **13**, 2259-2276. https://doi.org/10.5194/gmd-13-2259-2020 (2020)

12 Siewert, M. B. *et al.* Comparing carbon storage of Siberian tundra and taiga permafrost ecosystems at very high spatial resolution. *Journal of Geophysical Research: Biogeosciences* **120**, 1973-1994. https://doi.org/10.1002/2015JG002999 (2015)

13 Hersbach, H. *et al.* The ERA5 global reanalysis. *Quarterly Journal of the Royal Meteorological Society* **146**, 1999-2049. https://doi.org/10.1002/qj.3803 (2020)

14 GTN-P. https://gtnp.arcticportal.org/data/data-download (Akureyri, Iceland, 2016).

15 van Huissteden, J. *Thawing Permafrost: Permafrost Carbon in a Warming Arctic*. https://doi.org/10.1007/978-3-030-31379-1 (Springer Nature, 2020).

16 Li, B. *et al.* Thaw pond development and initial vegetation succession in experimental plots at a Siberian lowland tundra site. *Plant and Soil* **420**, 147-162. https://doi.org/10.1007/s11104-017-3369-8 (2017)

17 Nauta, A. L. *et al.* Permafrost collapse after shrub removal shifts tundra ecosystem to a methane source. *Nature Climate Change* **5**, 67-70, https://doi.org/10.1038/nclimate2446 (2015)

18 Blok, D. *et al.* Shrub expansion may reduce summer permafrost thaw in Siberian tundra. *Global Change Biology* **16**, 1296-1305, https://doi.org/10.1111/j.1365-2486.2009.02110.x (2010)

19 Magnusson, R. I. *et al.* Rapid Vegetation Succession and Coupled Permafrost Dynamics in Arctic Thaw Ponds in the Siberian Lowland Tundra. *Journal of Geophysical Research-Biogeosciences* **125**, https://doi:10.1029/2019jg005618 (2020).

20 Caswell, H. & Takada, T. Elasticity analysis of density-dependent matrix population models: the invasion exponent and its substitutes. *Theoretical Population Biology* **65**, 401-411. https://doi.org/10.1016/j.tpb.2003.09.007 (2004)

21 Benham, E., Ahrens, R. & Nettleton, W. Classification of Soil Texture Class Boundaries. https://www.nrcs.usda.gov/Internet/FSE_DOCUMENTS/nrcs142p2_031477.pdf (ed National Soil Survey Center) (USDA-NRCS, Lincoln, Nebraska, 2009).

22 USDA-ARS, U.S. Salinity Lab. ROSETTA Class Average Hydraulic Parameters. https://www.ars.usda.gov/pacific-west-area/riverside-ca/agricultural-water-efficiency-and-salinity-research-unit/docs/model/rosetta-class-average-hydraulic-parameters/ (ed Agricultural Water Efficiency and Salinity Research Unit) (U.S. DEPARTMENT OF AGRICULTURE Agricultural Research Service, Riverside, California, 2019).

23 Dalla Santa, G. *et al.* An updated ground thermal properties database for GSHP applications. *Geothermics* **85**, 101758, https://doi.org/10.1016/j.geothermics.2019.101758 (2020)

24 Jafarov, E. E. *et al.* Estimation of subsurface porosities and thermal conductivities of polygonal tundra by coupled inversion of electrical resistivity, temperature, and moisture content data. *The Cryosphere* **14**, 77-91. https://doi.org/10.5194/tc-14-77-2020 (2020)

25 Schuh, C., Frampton, A. & Christiansen, H. H. Soil moisture redistribution and its effect on inter-annual active layer temperature and thickness variations in a dry loess terrace in Adventdalen, Svalbard. *The Cryosphere* **11**, 635-651. https://doi.org/10.5194/tc-11-635-2017 (2017)

26 Soudzilovskaia, N. A., Bodegom, P. M. & Cornelissen, J. H. Dominant bryophyte control over high-latitude soil temperature fluctuations predicted by heat transfer traits, field moisture regime and laws of thermal insulation. *Functional Ecology* **27**, 1442-1454. https://doi.org/10.1111/1365-2435.12127 (2013)

27 Abramov, A. *et al.* Two decades of active layer thickness monitoring in northeastern Asia. *Polar Geography*, 1-17. https://doi.org/10.1080/1088937X.2019.1648581 (2019)
